# The interleukin-6 receptor as a target for prevention of coronary heart disease: a mendelian randomisation analysis

**DOI:** 10.1016/S0140-6736(12)60110-X

**Published:** 2012-03-31

**Authors:** The Interleukin-6 Receptor Mendelian Randomisation Analysis (IL6R MR) Consortium

## Abstract

**Background:**

A high circulating concentration of interleukin 6 is associated with increased risk of coronary heart disease. Blockade of the interleukin-6 receptor (IL6R) with a monoclonal antibody (tocilizumab) licensed for treatment of rheumatoid arthritis reduces systemic and articular inflammation. However, whether IL6R blockade also reduces risk of coronary heart disease is unknown.

**Methods:**

Applying the mendelian randomisation principle, we used single nucleotide polymorphisms (SNPs) in the gene *IL6R* to evaluate the likely efficacy and safety of IL6R inhibition for primary prevention of coronary heart disease. We compared genetic findings with the effects of tocilizumab reported in randomised trials in patients with rheumatoid arthritis.

**Findings:**

In 40 studies including up to 133 449 individuals, an *IL6R* SNP (rs7529229) marking a non-synonymous *IL6R* variant (rs8192284; p.Asp358Ala) was associated with increased circulating log interleukin-6 concentration (increase per allele 9·45%, 95% CI 8·34–10·57) as well as reduced C-reactive protein (decrease per allele 8·35%, 95% CI 7·31–9·38) and fibrinogen concentrations (decrease per allele 0·85%, 95% CI 0·60–1·10). This pattern of effects was consistent with IL6R blockade from infusions of tocilizumab (4–8 mg/kg every 4 weeks) in patients with rheumatoid arthritis studied in randomised trials. In 25 458 coronary heart disease cases and 100 740 controls, the *IL6R* rs7529229 SNP was associated with a decreased odds of coronary heart disease events (per allele odds ratio 0·95, 95% CI 0·93–0·97, p=1·53×10^−5^).

**Interpretation:**

On the basis of genetic evidence in human beings, IL6R signalling seems to have a causal role in development of coronary heart disease. IL6R blockade could provide a novel therapeutic approach to prevention of coronary heart disease that warrants testing in suitably powered randomised trials. Genetic studies in populations could be used more widely to help to validate and prioritise novel drug targets or to repurpose existing agents and targets for new therapeutic uses.

**Funding:**

UK Medical Research Council; British Heart Foundation; Rosetrees Trust; US National Heart, Lung, and Blood Institute; Du Pont Pharma; Chest, Heart and Stroke Scotland; Wellcome Trust; Coronary Thrombosis Trust; Northwick Park Institute for Medical Research; UCLH/UCL Comprehensive Medical Research Centre; US National Institute on Aging; Academy of Finland; Netherlands Organisation for Health Research and Development; SANCO; Dutch Ministry of Public Health, Welfare and Sports; World Cancer Research Fund; Agentschap NL; European Commission; Swedish Heart-Lung Foundation; Swedish Research Council; Strategic Cardiovascular Programme of the Karolinska Institutet; Stockholm County Council; US National Institute of Neurological Disorders and Stroke; MedStar Health Research Institute; GlaxoSmithKline; Dutch Kidney Foundation; US National Institutes of Health; Netherlands Interuniversity Cardiology Institute of the Netherlands; Diabetes UK; European Union Seventh Framework Programme; National Institute for Healthy Ageing; Cancer Research UK; MacArthur Foundation.

## Introduction

Inflammation is implicated in atherogenesis,^1^ but a causal association with a specific inflammatory mediator has not been established. Interleukin 6, an inflammatory cytokine produced mainly by T cells, macrophages, and adipocytes, promotes inflammatory responses via the membrane-bound or circulating soluble interleukin-6 receptor (IL6R) on monocytes, hepatocytes, and endothelial cells^2^ (appendix p 26). Similarly to C-reactive protein and fibrinogen, whose synthesis is stimulated by IL6R signalling, high circulating concentrations of interleukin 6 were associated with increased risk of coronary heart disease events in prospective observational studies.^3^, ^4^, ^5^ Despite exclusion of C-reactive protein and fibrinogen as causal mediators, on the basis of mendelian randomisation studies^6^, ^7^ IL6R signalling could be an important therapeutic target for prevention of coronary heart disease.

Tocilizumab, a monoclonal antibody that blocks both membrane-bound and circulating IL6R, has anti-inflammatory actions that extend beyond reductions in C-reactive protein and fibrinogen concentrations.^8^, ^9^ Tocilizumab is licensed for treatment of rheumatoid arthritis^10^, ^11^, ^12^ and has been shown to reduce articular inflammation and promote disease remission.^13^, ^14^ However, adequately powered, long-term trials of tocilizumab on risk of cardiovascular disease have not yet been undertaken.

Randomised trials in patients with rheumatoid arthritis revealed that tocilizumab increases total, HDL, and LDL cholesterol and triglycerides,^15^, ^16^ yet whether these lipid changes are on-target or off-target effects of tocilizumab, or whether they reflect a non-specific alleviation of suppressed inflammation as reported with other anti-inflammatory rheumatoid arthritis treatments, is uncertain.^17^ Whether the potentially proatherogenic increases in LDL cholesterol are offset by potentially antiatherogenic effects of reduced inflammation, or by the increase in HDL cholesterol, is also uncertain. Recognising that patients with rheumatoid arthritis are at increased risk of cardiovascular disease by virtue of their autoimmune disease and related vascular pathological changes^18^ and that tocilizumab is intended as a long-term treatment, the US Food and Drug Administration has mandated randomised controlled trials examining the cardiovascular effects of tocilizumab in patients with rheumatoid arthritis^12^ (NCT01331837 and NCT00535782). However, these ongoing trials will not answer the question of whether IL6R blockade will modify risk of coronary heart disease in the general population.

A recently developed extension to the mendelian randomisation paradigm^19^, ^20^—mendelian randomisation for drug target validation—uses variants in a gene encoding a drug target to profile the mechanism-based effects of pharmacological modification of that target and to distinguish on-target from off-target actions.^21^ By providing randomised evidence for the likely effectiveness of a new treatment in human beings without the potential risks of exposure to a novel drug or the cost of a randomised trial, this approach could aid prioritisation of targets for drug development. We applied mendelian randomisation to examine whether IL6R modulation is likely to reduce risk of coronary heart disease in the general population. We first evaluated the legitimacy of single nucleotide polymorphisms (SNPs) in the *IL6R* gene (Ch1q21.3) as indicators of the mechanism-based effect of pharmacological interference in IL6R signalling (appendix p 26). We then undertook a large-scale collaborative genetic association analysis of *IL6R* variants with coronary heart disease events and stroke and examined safety endpoints, including infections and common cancers.

## Methods

### Treatment trials and other studies of tocilizumab

Following PRISMA guidelines^22^ (appendix p 28), we searched Medline using PubMed for randomised trials, cohorts, or meta-analyses comparing tocilizumab (4 or 8 mg/kg) with placebo in human beings (appendix pp 1, 7). Details of extracted data and methods used to synthesise and combine estimates of trial results are reported in the appendix.

### Genetic association studies

We included individual participant data for up to 133 449 participants of European ancestry from 40 studies (appendix pp 1–7, 11–12). Data from this de-novo analysis were pooled with previously published information about the association of *IL6R* variants with clinical events. We gathered phenotypic data across several studies (appendix pp 13–14) for analysis of associations between *IL6R* genotype and interleukin 6, C-reactive protein, fibrinogen, and major blood lipid fractions.

The primary event endpoint for the genetic analysis was all fatal and non-fatal coronary heart disease events (consisting of myocardial infarction and coronary revascularisation; appendix pp 15–16). Secondary efficacy endpoints were all-cause stroke, and all fatal and non-fatal cardiovascular disease (consisting of myocardial infarction, coronary revascularisation, and stroke). These disease outcomes are analogous to the efficacy outcomes in an orthodox randomised trial.

On the basis of published associations of interleukin-6 concentrations with disease outcomes other than coronary heart disease, safety endpoints reported in tocilizumab trials, and standard safety endpoints for cardiovascular intervention trials, we investigated the association of *IL6R* variants with all-site cancer, major cancer subtypes (breast and colorectal), respiratory infection, and liver enzyme concentrations. We obtained safety data from de-novo investigations of *IL6R* variants, from estimates of the association of any safety outcome with *IL6R* variants reported in the National Human Genome Research Institute genome-wide association study catalogue,^23^ and from other reported associations of published genome-wide association studies (appendix p 17). We also estimated the association between *IL6R* variants and other established risk factors for cardiovascular disease including blood pressure and type 2 diabetes.

### SNP selection, genotyping, and quality control

Using the HumanCVD BeadChip,^24^ we genotyped 4489 individuals of European ancestry in the Whitehall II study. From the 42 SNPs located within 55 kb of *IL6R* present on the array, we selected a subset of SNPs for further analysis in other datasets on the basis of four factors: (1) the statistical strength of association with interleukin-6 concentration; (2) linkage disequilibrium (LD) between SNPs in populations of European ancestry using Human HapMap Phase 3 Build 36 data, to reduce redundancy; (3) previous disease and biomarker associations of SNPs in this region (appendix p 27); and (4) a minor allele frequency (MAF) threshold of greater than 0·3. Data were excluded if the allele call rate was less than 90% or the Hardy-Weinberg equilibrium (HWE) χ^2^ p value was less than 0·001 in any study.

### Statistical analysis

Genotypes were coded as 0, 1, and 2, indicating the number of variant allele copies. The analysis was done with an additive model suggested by *IL6R* associations with circulating interleukin-6 concentrations in the index Whitehall II Study. Owing to skewed distributions, values of interleukin 6, C-reactive protein, fibrinogen, and triglycerides were analysed on the natural logarithmic scale.

Using individual participant-level data, we estimated the mean difference in interleukin 6, C-reactive protein, and fibrinogen between genotype groups for each SNP. Additionally, for these inflammatory markers, the major lipid fractions, and other biomarkers, we fitted univariate linear regression models within each study to investigate evidence of a linear association between the biomarker and possession of each additional copy of the minor allele. All analyses were done within each study according to a common analysis plan implemented with a standardised Stata (version 11.1) program, adapted for SPSS and PLINK in some studies. Where possible, we repeated analyses in prespecified subgroups (appendix pp 5–6).

To assess association of SNPs with disease endpoints, we estimated unadjusted odds ratios (OR) per minor allele within each study using logistic regression models. In studies for which relevant data were available (27 studies, 97 300 participants), we estimated the per-allele OR for coronary heart disease events, stratified where appropriate by prespecified characteristics (appendix pp 5–6) within each study, and by study design. Within-study estimates were combined with inverse-variance weighted fixed-effects meta-analysis. We used *I*^2^ to quantify between-study heterogeneity.^25^ In subgroup analyses, we tested for heterogeneity between strata using the meta-analysis χ^2^ test for heterogeneity.

### Role of the funding source

The funding sources had no role in study design, in the collection, analysis, and interpretation of data, in the writing of the report, or in the decision to submit for publication. The corresponding author (DIS) and co-senior authors (ADH and JPC) had full access to all data in the study and had final responsibility for the decision to submit for publication.

## Results

We identified six short-term randomised trials (12–52 week duration) evaluating 4 mg/kg or 8 mg/kg tocilizumab in 2891 patients with rheumatoid arthritis (weighted mean age 52·3 years; 19% male; appendix p 28).^8^, ^9^, ^26^, ^27^, ^28^, ^29^ C-reactive protein was the most widely reported inflammation marker and its weighted mean concentration at baseline was 28·2 (SD 1·9) mg/L. Tocilizumab treatment (4, 8, and 16 mg/kg in randomised or observational studies every 4 weeks) was associated with a dose-dependent reduction in C-reactive protein concentration (appendix p 29). The 8 kg/mg dose reduced fibrinogen, increased interleukin 6 and soluble IL6R, and increased LDL and HDL cholesterol (table).

**Table:** Summary effects of tocilizumab (8 mg/kg) and the *IL6R* rs7529229 variant on inflammatory, lipid, hepatic, and haematological biomarkers

	**Randomised trials of tocilizumab (8 mg/kg)**			**Genetic studies (present analyses; per-allele effect)**		
	Number of individuals (trials)	Summary effect (95% CI)	p value	Number of individuals (studies)	Summary effect (95% CI)	p value
**Mean difference**						
Interleukin 6 (pg/mL)	1446 (4)	28·89 (23·04 to 34·75)	4·10×10^−22^	29 838 (17)	0·09 (0·08 to 0·10)*	8·41×10^−68^
Soluble IL6R (ng/mL)	1465 (4)	529·87 (529·29 to 530·45)	<1×10^−95^	1454 (3)	14·87 (13·07 to 16·66)	2·69×10^−59^
C-reactive protein (mg/L)	3010 (6)	–19·02 (–16·28 to −21·72)	2·37×10^−37^	76 527 (30)	–0·09 (–0·10 to −0·08)*	9·92×10^−52^
Fibrinogen (g/L)	108 (1)	–2·50 to (–2·50 to −3·11)	7·14×10^−16^	52 667 (19)	–0·009 (–0·011 to −0·006)*	3·25×10^−11^
Total cholesterol (mmol/L)	955 (4)	0·89 (0·78 to 0·99)	2·81×10^−42^	114 615 (33)	0·004 (–0·005 to 0·013)	0·37
HDL cholesterol (mmol/L)	616 (3)	0·12 (0·07 to 0·17)	4·02×10^−6^	105 439 (30)	0·002 (–0·001 to 0·006)	0·18
LDL cholesterol (mmol/L)	409 (1)	0·57 (0·45 to 0·69)	7·41×10^−22^	97 966 (28)	–0·003 (–0·012 to 0·005)	0·45
Triglycerides	··	··	··	105 656 (30)	–0·002 (–0·006 to 0·002)*	0·42
Albumin (g/L)	108 (1)	6·00 (4·50 to 7·50)	6·55×10^−15^	5787 (3)	0·10 (0·01 to 0·20)	0·03
ESR (mm/h)	1658 (4)	–30·49 (–27·83 to −33·14)	5·55×10^−95^	··	··	··
Platelets (×10^9^/L)	108 (1)	–1·27 (–1·66 to −0·88)	1·72×10^−10^	3274 (1)	–0·76 (–4·33 to 2·81)	0·68
Serum amyloid A†	517 (2)	–0·75 (–0·57 to −0·93)	1·28×10^−16^	··	··	··
Haemoglobin (g/L)	2072 (4)	12·7 (11·1 to 14·2)	1·96×10^−56^	17 898 (4)	0·022 (0·002 to 0·043)	0·04
AST (U/L)	··	··	··	7201 (4)	0·006 (–0·003 to 0·016)	0·20
**Odds ratio**						
Triglycerides >5·7 mmol/L	1220 (1)	1·42 (0·42 to 4·42)	0·55	··	··	··
ALT 3×ULN	2755 (4)	6·95 (3·58 to 13·50)	9·95×10^−9^	··	··	··
AST 3×ULN	2420 (3)	4·74 (1·66 to 13·62)	0·004	··	··	··

Summary effect is mean difference (95% CI) for all biomarkers apart from triglycerides greater than 5·7 mmol/L (500 mg/L), ALT 3×ULN, and AST 3×ULN, for which estimates are odds ratio (95% CI). For tocilizumab, the mean difference is for tocilizumab versus placebo and for the *IL6R* rs7529229 variant, the mean difference is per minor allele. Trial data are for comparison of tocilizumab (8 mg/kg daily) and placebo groups at timepoints between 6 and 24 weeks (apart from C-reactive protein, which was taken at 8 weeks for all trials except one,^9^ which reported values at 52 weeks). IL6R=interleukin-6 receptor. ESR=erythrocyte sedimentation rate. ALT=alanine transaminase. ULN=upper limit of normal. AST=aspartate transaminase.

* Mean difference per minor allele on the log_e_ scale represents proportional difference in geometric mean.

† For serum amyloid A, we could not harmonise units, thus the standardised mean difference is presented.

40 studies contributed genotype and phenotype data for the de-novo genetic analysis of *IL6R* SNPs from a total of 133 449 individuals with mean age at recruitment of 59 (range 26–75) years, of whom 49% were women. Additional characteristics of study participants are described in appendix pp 11–16, 30–33. 12 of the 42 SNPs in the region of the *IL6R* locus on the HumanCVD BeadChip met chip-wide significance (p<1×10^−5^) for their association with circulating interleukin 6 in the Whitehall II study (appendix pp 20–21). We selected three SNPs (rs7529229, rs4845371, rs12740969) based on MAF greater than 0·3, β coefficient greater than 0·9 log interleukin-6 concentration per allele, previously reported associations, and low-redundancy LD structure (appendix p 27). The rs7529229 variant was in strong LD (*r*^2^=0·92 in the Whitehall II study) with a non-synonymous variant (rs8192284, also annotated as rs2228145, which did not meet our initial selection criteria) previously reported to be associated with increased proteolytic cleavage of the soluble IL6R from its membrane-bound form^30^, ^31^ (see appendix p 26 for mechanistic details) and became our lead SNP for the analysis. Where rs7529229 was not genotyped, a proxy SNP was used (defined on the basis of *r*^2^≥0·90 with rs7529229 in individuals of European ancestry; appendix pp 18–19). Information about rs4845371 and rs12740969 is reported in subsidiary analyses. 40 studies (133 449 participants) provided data for rs7529229, 18 studies (52 475 participants) for rs4845371, and 19 studies (59 126 participants) for rs12740969. All studies met the prespecified quality control threshold criteria for call rate, HWE, and MAF (appendix pp 18–19).

**Figure 1:**
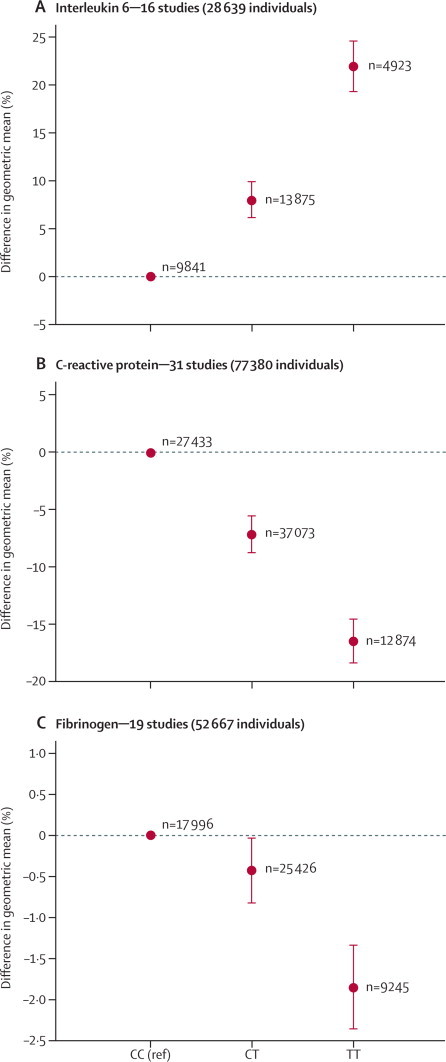
Association of the *IL6R* rs7529229 variant with (A) interleukin 6, (B) C-reactive protein, and (C) fibrinogen concentration Estimates are based on pairwise comparison of individuals heterozygous or homozygous for the variant T allele with reference to the CC homozygous group. The total number of studies and participants are also shown. Error bars show 95% CIs.

The *IL6R* rs7529229 SNP displayed additive associations with circulating concentrations of interleukin 6, C-reactive protein, and fibrinogen (figure 1, table). Circulating interleukin-6 concentration increased with each additional copy of the minor allele at rs7529229 (relative increase in geometric mean log interleukin-6 concentration per allele 9·45%, 95% CI 8·34–10·57; p=8·41×10^−68^), whereas the concentrations of C-reactive protein and fibrinogen decreased per minor allele (relative decrease in geometric mean log C-reactive protein 8·35%, 95% CI 7·31–9·38, and fibrinogen 0·85%, 0·60–1·10, per minor allele). The associations with interleukin 6 and C-reactive protein were consistent across study-specific subgroups (appendix pp 34–37) with no evidence of genotype-by-subgroup interaction (p>0·05 for all analyses). Concentration of soluble IL6R increased per minor allele (table). The functional rs8192284 variant showed associations with interleukin 6, C-reactive protein, and fibrinogen that were directionally concordant with those of rs7529229 in the Whitehall II study (appendix p 24). No significant association was noted between the rs7529229 SNP and concentration of total, LDL, and HDL cholesterol or triglycerides in analyses including up to 114 615 individuals (table).

**Figure 2:**
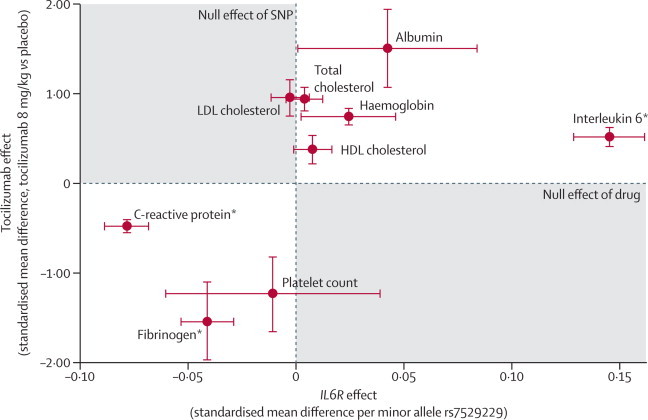
Associations of the minor allele of the *IL6R* SNP rs7529229 and tocilizumab (8 mg/kg) versus placebo with commonly reported biomarkers Concordance between the drug and genetic variants is shown. Effects are presented as standardised mean difference apart from log_e_ transformed variables (shown by *), for which rs7529229 effects represent the mean difference on the log scale. Estimates for soluble interleukin-6 receptor were not plotted since their substantially greater magnitude would disrupt the scale of the graph: standardised mean differences were 0·75 (95% CI 0·59–0·91) ng/mL per minor allele for rs7529229, and 93·67 (95% CI 90·27–97·06) ng/mL for tocilizumab 8 mg/kg versus placebo. SNP=single nucleotide polymorphism.

The blood markers interleukin 6, soluble IL6R, C-reactive protein, fibrinogen, and total, LDL, and HDL cholesterol were available in both genetic studies and tocilizumab treatment trials allowing a direct comparison of *IL6R* genotype and IL6R blockade (table). The minor allele of rs7529229 and treatment with tocilizumab showed directionally concordant effects; each was associated with reduced C-reactive protein and fibrinogen and increased interleukin 6 and soluble IL6R (table, figure 2). Tocilizumab treatment increased circulating total, HDL, and LDL cholesterol, and triglycerides, but the *IL6R* rs7529229 SNP, by contrast, showed no significant association with any of these lipid fractions (table, figure 2). In randomised trials, tocilizumab increased concentrations of albumin and haemoglobin and decreased erythrocyte sedimentation rate (ESR), platelet count, and serum amyloid A (table). The effect of rs7529229 was directionally concordant with that of tocilizumab on albumin, haemoglobin, and platelet count (table, figure 2). Data for ESR were unavailable in the genetic studies, but plasma viscosity (reflected by ESR) was lower in carriers of the rs7529229 minor allele (mean difference per allele −2·16×10^−3^ mPa.s, 95% CI −3·86×10^−4^ to −3·94×10^−3^, p=0·02; five studies, 15 589 individuals). Absence of data for serum amyloid A in the genetic analysis precluded comparison with tocilizumab treatment. In comparison of tocilizumab treatment with the rs7529229 variant, the direction of effect was concordant for nine of the ten biomarkers (table, figure 2), and greater than expected under the null hypothesis of no concordance (binomial test, p=0·01).

**Figure 3:**
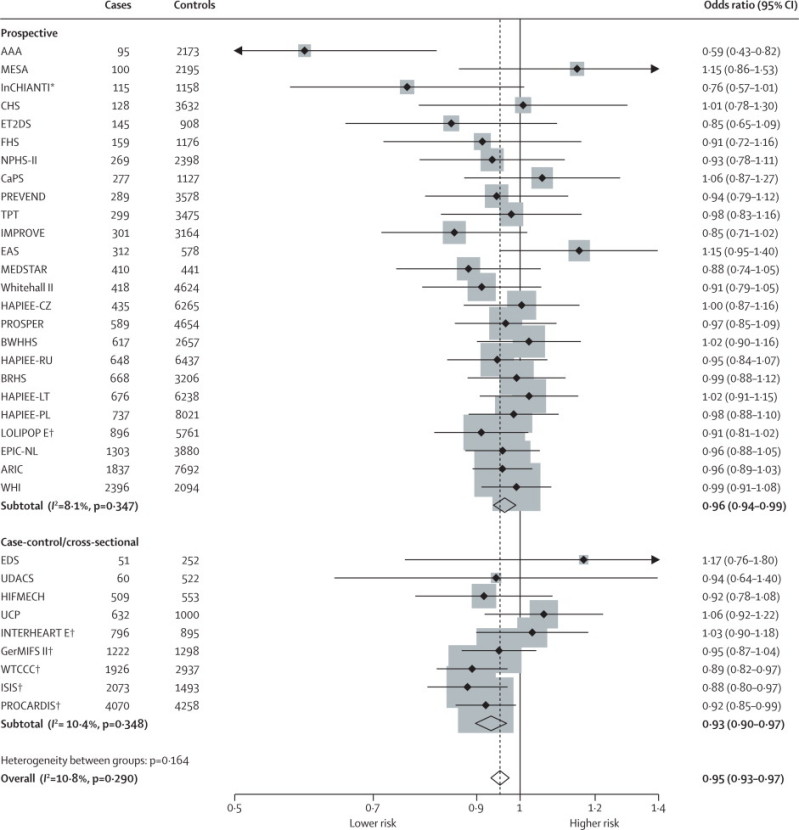
Association of *IL6R* rs7529229 with risk of fatal and non-fatal coronary heart disease Individual study odds ratios were based on a per-allele model and pooled with fixed effects meta-analysis. *Data published in reference 30. †Data published in reference 32.

We also examined the association of *IL6R* variants with coronary heart disease. In a meta-analysis of 34 studies (25 458 coronary heart disease cases, 100 740 controls) the OR for the primary outcome (all fatal and non-fatal coronary heart disease events; appendix pp 15–16) per minor allele at rs7529229 was 0·95 (95% CI 0·93–0·97, p=1·53×10^−5^). There was low heterogeneity between studies (*I*^2^=10%, 95% CI 0–41) and the effect estimates were consistent in prospective and case-control studies, including previously published data^32^ (figure 3). In a subset of 97 300 individuals (27 studies) for whom relevant data were available, the association of rs7529229 with the primary outcome (14 360 cases and 82 940 controls) was consistent in stratified analyses (appendix pp 1–7) with no evidence for effect modification by any of these subgroups (appendix pp 40–41).

**Figure 4:**
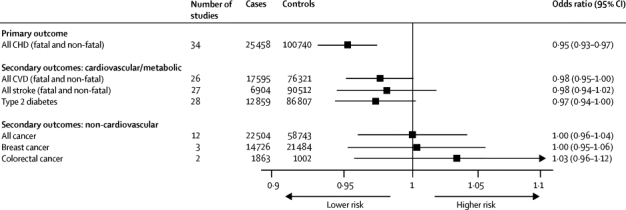
Association of *IL6R* rs7529229 with secondary and safety endpoints Summary per-allele odds ratio for cardiovascular and non-cardiovascular endpoints for the *IL6R* rs7529229 variant. Individual study odds ratios were based on a per-allele model in the present collaborative analysis and genome-wide association studies and pooled with fixed effects meta-analysis. CHD=coronary heart disease. CVD=cardiovascular disease.

Associations of rs7529229 with risk of fatal or non-fatal stroke (OR 0·98, 95% CI 0·95–1·02, p=0·30) in 6904 cases and 90 512 controls (27 studies) and with fatal or non-fatal cardiovascular disease events combined (OR 0·98, 95% CI 0·95–1·00, p=0·05) in 17 595 cases and 76 321 controls (26 studies) were suggestive but not compelling (figure 4). Up to three of six randomised trials of tocilizumab reported the incidence of cardiac or vascular events, or both, with median follow-up of 24 weeks (appendix pp 22–23). However, imprecise endpoint definition and the small number of events prevented comparison with genetic studies.

In an analysis of safety endpoints in tocilizumab trials, data suggested an increased risk of infection (OR 1·30, 95% CI 1·07–1·58) and increased concentrations of hepatic enzymes alanine transaminase and aspartate transaminase (table) with tocilizumab treatment compared with placebo (appendix pp 22–23). By contrast with evidence for tocilizumab, genetic analyses (although in a relatively small subset) did not reveal any association with concentrations of aspartate transaminase (table) or in log γ-glutamyl transferase (relative difference in geometric mean per allele −0·64%, 95% CI −1·95 to 0·69, p=0·34; seven studies, 15 641 individuals). Our genetic experiment did not include infection as an outcome and published evidence for the *IL6R* rs7529229 variant was scarce.^33^ Genome-wide association studies of tuberculosis^34^ and meningococcal disease^35^ have not reported associations of variants in *IL6R* with risk of those outcomes.

Neither the evidence from tocilizumab trials nor the genetic studies to date have suggested an association of IL6R blockade with increased risk of cancer. The pooled OR for development of any cancer was 0·42 (95% CI 0·06–2·88; four cases and 1196 controls) for tocilizumab treatment in randomised trials, and was 0·98 (95% CI 0·93–1·03; 5376 cases and 57 123 controls) for the *IL6R* rs7529229 variant. In published genome-wide association studies and new look-ups, the *IL6R* rs7529229 variant showed no association with breast cancer (OR 1·01, 95% CI 0·94–1·10) or colorectal cancer (OR 1·03, 95% CI 0·96–1·12; figure 4; appendix p 17).

The *IL6R* rs7529229 variant was associated with lowered systolic blood pressure (per-allele β coefficient −0·21 mm Hg, 95% CI −0·37 to −0·05, p=0·01) and diastolic blood pressure (per-allele β coefficient −0·11 mm Hg, 95% CI −0·20 to −0·02, p=0·02) in 33 studies (112 979 individuals). There was suggestive evidence that the rs7529229 variant was associated with reduced risk of type 2 diabetes (OR 0·97, 95% CI 0·94–1·00, p=0·06) in 12 859 cases and 86 807 controls (figure 4), although this exploratory finding needs further investigation.

## Discussion

Our study provides strong evidence in human beings for a causal role of a specific inflammatory mechanism (ie, IL6R signalling) in coronary heart disease (panel). A common polymorphism in *IL6R* marking a non-synonymous variant (p.Asp358Ala) with known functional consequences^30^, ^36^ was associated with differences in circulating concentrations of soluble IL6R, interleukin 6, C-reactive protein, and fibrinogen that were directionally concordant with those reported in trials of IL6R blockade with tocilizumab. Meta-analysis of 34 studies including 25 458 coronary heart disease cases and 100 740 controls suggested the same *IL6R* rs7529229 variant was associated with reduced odds of coronary heart disease events. This finding suggests that targeting of IL6R could provide a novel therapeutic approach to prevention of coronary heart disease.

Panel **Research in context**

**Systematic review**

We searched Medline via PubMed for “tocilizumab AND (“coronary heart disease” OR “myocardial infarction”), for “interleukin-6 receptor AND (“coronary heart disease” OR “myocardial infarction”), and for “IL6R” AND (“coronary heart disease” OR “myocardial infarction”) up to Jan 8, 2012. Local and systemic inflammation is implicated in atherosclerosis but, as yet, there are no licensed approaches for prevention of cardiovascular disease that target inflammatory mechanisms. Mendelian randomisation studies suggest that two intensively studied biomarkers of inflammation associated with cardiovascular disease, C-reactive protein and fibrinogen, are unlikely to be causally related to atherosclerosis. High circulating interleukin-6 concentration is associated with increased risk of coronary heart disease in observational studies and preliminary evidence suggests a variant in the gene for its receptor (*IL6R*) might be associated with reduced risk of coronary heart disease.^31^ Tocilizumab, a monoclonal antibody targeting the interleukin-6 receptor (IL6R), is licensed for treatment of rheumatoid arthritis, but whether IL6R blockade reduces risk of coronary heart disease is unknown.

**Interpretation**

Applying the mendelian randomisation principle, we found that a variant in the *IL6R* gene had effects on biomarkers of inflammation and related pathways (including interleukin 6, C-reactive protein, fibrinogen, haemoglobin, albumin, and others) that are directionally concordant with those of tocilizumab treatment reported by randomised trials. In keeping with that expected for common alleles, the size of the genetic effect was small compared with that of the drug. Nevertheless, the same genetic variant was associated with a lowered risk of coronary heart disease in a sample of 25 458 coronary heart disease cases and 100 740 controls (odds ratio per minor allele 0·95, 95% CI 0·93–0·97, p=1·53×10^−5^). This mendelian randomisation analysis suggests that IL6R signalling is involved in coronary heart disease and that the IL6R could be a valuable target for the prevention or treatment of coronary heart disease.

Although the *IL6R* rs7529229 variant was associated with reduced circulating C-reactive protein and fibrinogen concentrations, this study should not be interpreted as a mendelian randomisation analysis investigating causality of C-reactive protein or fibrinogen in coronary heart disease. Previous large mendelian randomisation studies using SNPs in the genes encoding C-reactive protein and fibrinogen^6^, ^7^, ^32^, ^37^, ^38^, ^39^ suggested that neither is a causal mediator of coronary heart disease. Therefore, other consequences of reduced interleukin-6 signalling could be responsible for the association with decreased risk of coronary heart disease that we identified. Specific mendelian randomisation analyses for interleukin 6 have not yet been done, largely because SNPs in the gene encoding interleukin 6 (*IL6*, Ch7p15.3) that reliably associate with circulating interleukin-6 concentration have not been identified.^40^, ^41^ By contrast, the present study provides an example of a different type of mendelian randomisation analysis: one used to validate a drug target.

Although the association of the *IL6R* variant with raised concentrations of interleukin 6 and reduced coronary risk noted in this study might seem paradoxical, the pattern is consistent with pharmacological blockade of IL6R with tocilizumab. The finding can be explained by reduced IL6R signalling in carriers of the variant allele, which leads to attenuation of downstream consequences of interleukin 6 (of which a reduction in C-reactive protein and fibrinogen concentrations are but two), and an accumulation or release of feedback inhibition of the upstream ligand (interleukin 6) and its soluble receptor.^42^

Randomised trials of tocilizumab in patients with rheumatoid arthritis reported increases in blood lipid fractions.^15^, ^16^ By contrast, carriage of the *IL6R* rs7529229 minor allele was not associated with changes in any major blood lipid fraction. Evidence suggesting individuals carrying the rs7529229 variant were more likely to use lipid-lowering drugs than were non-carriers was weak (OR per minor allele 1·02, 95% CI 0·99–1·06, p=0·24); the absence of association with blood lipids was consistent between users and non-users of these drugs (heterogeneity χ^2^ p=0·15).

There are several potential explanations for the discordance in effects on blood lipids between tocilizumab treatment and *IL6R* genotype. First, randomised trials of tocilizumab were done in patients who had higher levels of background inflammation (baseline mean C-reactive protein 28·4 mg/L) than did participants in the genetic studies sampled from general populations (geometric mean C-reactive protein 1·8 mg/L). The effects of tocilizumab on lipids might be mechanism-based but only manifest on a background of substantial systemic inflammation (which, in many conditions, is associated with reduced circulating lipid concentration^43^) and therefore not detectable at low levels of inflammation seen in healthy individuals. Second, there might be differences between the lifelong effect of genetic variants in *IL6R* and the short-term, later-life exposure to tocilizumab. Third, the effect of pharmacological blockade and genetic variation on classic signalling through the membrane-bound IL6R versus trans-signalling via the soluble receptor might also differ. Tocilizumab binds both the soluble and membrane-bound receptors inhibiting classical and trans pathways,^44^, ^45^ but the functional polymorphism tagged by rs7529229 (rs8192284) results in increased soluble IL6R concentration through increased proteolytic cleavage of the membrane-bound receptor^46^, ^47^, ^48^ and possibly a reduction in the number of functioning membrane-bound receptors.^30^, ^36^ Membrane-bound IL6R mediates interleukin-6 signalling in hepatocytes and some leucocyte populations, whereas the soluble receptor acts on a diverse range of cell types including megakaryocytes and endothelial cells;^2^, ^48^ both mechanisms rely on the ubiquitously expressed signal transducer, gp130. IL6R-mediated effects on blood lipids might also need a suprathreshold change in IL6R signalling, which might be achieved by pharmacological inhibition but not by natural genetically-mediated changes in the concentration or function of the IL6R. Finally, the possibility remains that the lipid-related effects of tocilizumab are an off-target action.

The association of the *IL6R* rs7529229 variant with lowered risk of coronary heart disease provides robust evidence of a role for inflammation in pathogenesis of coronary heart disease that is consistent with previously reported findings based on the *IL6R* rs4537545 SNP (in LD with rs7529229, *r^2^*=1·00),^32^ although our analysis included more than twice the number of cases. The effect estimates obtained from our de-novo analysis of largely prospective studies and the previous analysis based mainly on case-control studies were highly consistent with no evidence of heterogeneity in the effect estimates obtained from prospective, case-control, or cross-sectional studies. Furthermore, we did not identify heterogeneity in the genetic effects in individuals stratified by prevailing concentrations of non-HDL cholesterol or by lipid-lowering drug use, generating the hypothesis that the effects of IL6R blockade could be additive to those of established lipid-based interventions.

The randomised trials of tocilizumab designed to examine drug efficacy in rheumatoid arthritis were fairly small and of short duration. Cardiac and vascular safety endpoints were reported as part of the safety assessment, but only one trial^28^ reported myocardial infarctions. Absence of detail on the definitions of safety outcomes in the remaining randomised trials made assessment of the effect of tocilizumab on risk of coronary events too imprecise to be valuable. Infections were the most commonly reported adverse events in tocilizumab trials.^15^ We lacked data for infectious events in the genetic studies; however, published evidence from candidate gene studies have not suggested an *IL6R* association with risk of respiratory infection.^33^ Risk of incident infection would be an important safety consideration in any trial of IL6R inhibition for prevention of coronary heart disease.

In addition to increases in blood lipids, there were infrequent reports of raised hepatic enzymes in trials of tocilizumab, although the magnitude of these changes did not increase with prolonged exposure.^15^ We noted no association of the lead *IL6R* SNP with aspartate transaminase. Analysis of data from genetic studies in this collaboration and those reported in the literature (including genome-wide association studies and other large-scale studies) did not reveal increased risk of common cancers. Our safety profiling of IL6R blockade in the genetic experiment included the effect of *IL6R* variants on established cardiovascular risk factors such as type 2 diabetes and blood pressure. The findings were suggestive of associations of the rs7529229 SNP with reduced risk of type 2 diabetes and lowered systolic and diastolic pressures, although these need further investigation.

This large-scale analysis provides reliable genetic evidence for the role of a specific inflammatory pathway in the development of coronary heart disease in humans. Comparison of the genetic findings with data from randomised trials of tocilizumab supports further evaluation of IL6R inhibition as a therapeutic strategy for prevention of coronary heart disease. Other monoclonal antibodies against IL6R are now in advanced development and small molecules with activity at IL6R have also been reported.^49^ An ongoing trial of the anti-interleukin-1β monoclonal antibody canakinumab for reduction of coronary heart disease risk (NCT01327846) underlines the potential of inflammatory pathways as targets for cardiovascular prevention and supports a need for a trial of IL6R inhibition for prevention of coronary heart disease events.

Correspondence to: Dr Daniel I Swerdlow, Genetic Epidemiology Group, Research Department of Epidemiology and Public Health, UCL Institute of Epidemiology and Health Care, University College London WC1E 6BT, UK

d.swerdlow@ucl.ac.uk
