# Supplementary material for: The interleukin-6 receptor as a target for prevention of coronary heart disease: a mendelian randomisation analysis
Source: Lancet. 2012 Mar 31;379(9822):1214–24. doi: 10.1016/S0140-6736(12)60110-X (PMC3316968; doi:10.1016/S0140-6736(12)60110-X)
Supplement: Supplementary appendix [file mmc1.pdf]

THE LANCET

Supplementary appendix

This appendix formed part of the original submission and has been peer reviewed. We post it as supplied by the authors.

Supplement to: The Interleukin-6 Receptor Mendelian Randomisation Analysis (IL6R MR) Consortium. The interleukin-6 receptor as a target for prevention of coronary heart disease: a mendelian randomisation analysis. *Lancet* 2012; published online March 14. DOI:10.1016/S0140-6736(12)60110-X.

The interleukin-6 receptor as a target for prevention of coronary heart disease: a mendelian randomisation analysis

Supplementary Material

Supplementary methods

Treatment trials and other studies of tocilizumab

We restricted the main analysis to RCTs comparing tocilizumab treatment (4mg/kg or 8mg/kg) with placebo, with or without background treatment using other anti-inflammatory or immunosuppressive interventions. We also investigated the association between tocilizumab treatment and CRP concentration in non-randomised cohort studies.

Genetic studies

Study characteristics of genetic studies included in the analysis are detailed in **Supplementary table 1** . The studies from which individual participant data were included in the present analyses are listed below, and include 26 prospective studies, four randomised cardiovascular prevention trials, two cross-sectional studies, eight retrospective case-control studies.

Prospective cohort studies

Twenty-five prospective studies contributed genotypic and phenotypic data, and their design is described elsewhere.

From the UCL-LSHTM-Edinburgh-Bristol (UCLEB) Consortium:

British Regional Heart Study (BRHS)[1,] the British Women's Heart and Health Study (BWHHS)[2], the Caerphilly Prospective Study (CaPS)[3], the Edinburgh Artery Study (EAS)[4], the English Longitudinal Study of Aging (ELSA)[5], the Edinburgh Type 2 Diabetes Study (ET2DS)[6], the Northwick Park Heart Study II (NPHS-II)[7], the Whitehall-II (WHII) study[8], and the MRC National Study of Health and Development (1946 Birth Cohort, MRC NSHD)[9].

From the National Heart, Lung and Blood Institute (NHLBI) Candidate Gene Association Resource (CARe) Consortium[10]: the Atherosclerosis Risk in Communities Study (ARIC)[11], the Coronary Artery Risk

Development in Young Adults (CARDIA)[12], the Cleveland Family Study (CFS)[13,14], the Cardiovascular Health Study (CHS)[15], the Framingham Heart Study (FHS)[16], and the Multi-Ethnic Study of Atherosclerosis (MESA)[17].

From the Health, Alcohol and Psychosocial factors In Eastern Europe (HAPIEE) study[18]: the Czech Republic (HAPIEE-CZ), Lithuania (HAPIEE-LT), Poland (HAPIEE-PL) and Russia (HAPIEE-RU) arms of the study.

Also, the MRC Ely Cohort[19](40), the MRC Fenland Cohort (2 samples) [20], the IMPROVE study[21], the Prevention of Renal and Vascular Endstage Disease study (PREVEND)[22], the Second Manifestations of Arterial Disease (SMART) Study[23], the InCHIANTI study[24] and the MedStar study[25].

Three RCT studies were included - the Aspirin in Asymptomatic Atherosclerosis (AAA) trial[26], the PROSPER trial follow-up study[27], and the Thrombosis Prevention Trial follow-up study (TPT)[28]. These trials were treated as prospective cohorts for the purposes of these analyses.

Case-control, case-cohort and cross-sectional studies

Four nested case-control studies were included - the EPIC-Netherlands study (EPIC-NL)[29], the EPIC-Norfolk study (EPIC-N, case-cohort design)[30], the Utrecht Cardiovascular Pharmacogenetics study (UCP)[31] and the Women's Health Initiative (WHI)[32].

Two cross-sectional studies of diabetes were included - the Ealing Diabetes Study (EDS)[33] and the UCL Diabetes and Cardiovascular Study (UDACS)[34].

Four case-control and case-cohort studies contributed data: i) the Hypercoagulability and Impaired Fibrinolytic function Mechanisms (HIFMECH) study[35] included recent survivors of first myocardial infarction (MI) and age-matched healthy control;

ii) The Ischemic Stroke Genetics Study-Siblings With Ischaemic Stroke (ISGS-SWISS) consortium, a case-control GWAS study of ischaemic stroke[36,37];

iii) the Colorectal Tumour Gene Identification study (CORGI) [38]; and

iv) the Colorectal Cancer Genetics Study (COGS) - both GWA studies, contributed data on colorectal cancer. Further details of these studies are given in **Supplementary table 1**.

Genome-wide association studies and other cohorts contributing published, summary data

Thirteen published GWA studies contributed look-up data to the analyses. The GerMIFS II study[39], the INTERHEART study[40], the ISIS study[41,42], the LOLIPOP study[43], the PROCARDIS study[44], the Wellcome Trust Case-Control Collaboration[45], the British Breast Cancer Study[46], the Cancer Genetic Markers of Susceptibility study[47], the Nurses' Health Study, the National Cancer Research Network, Mammography, Oestrogens and Growth Factors study, the Colorectal Tumour Gene Identification study (CORGI) [38], and the MRC British 1958 Birth Cohort[48] contributed 13574 aggregated breast cancer cases and 14852 controls[49].

Phenotypic measures

Demographic, anthropometric and biomarker variables

Blood lipid fractions evaluated as primary biomarker outcomes were total, LDL- and HDL-cholesterol and triglycerides. Availability of data for biomarkers in the primary analysis is detailed in **Supplementary Table 2**. All biomarkers were measured using validated assays and protocols reported previously. Biomarker measurements were taken from either the baseline phase of each study or from the next phase soonest after baseline at which data on the greatest number of biomarkers were available. Where key biomarkers were unavailable at the principal survey phase their data were included from the chronologically closest available phase. Continuous variables that were not normally distributed were $\log_e$ transformed to achieve normality, and all biomarkers were converted to common units of measurement. Levels of LDL were not measured directly in some studies (HAPIEE-RU and CaPS) and were therefore calculated from data on total and HDL-cholesterol and triglyceride levels using the Friedewald formula[50].

Where data for LDL-, HDL- and total cholesterol were available in units of mg/dL, they were converted to mmol/L using a multiplication factor of 0.02586. Triglyceride units were similarly converted using a factor of 0.01129.

We also estimated *IL6R* SNP associations with haemoglobin, platelet count and hepatic enzyme levels that have also been reported to be altered by tocilizumab treatment[9,10,24–26].

Clinical outcome reporting

Since event definitions differed between collaborating studies, common definitions were set to accommodate data from as many cohorts as possible. Several studies reported validated events (using case-note data or

similar), but others recorded only self-reported events. We included all events in the analysis - self-reported and validated - since self-reported data was the lowest common denominator between studies (**Supplementary Table 3**). Where possible, only validated self-reported events were included. The primary outcome (all fatal and non-fatal CHD events) included acute myocardial infarction (MI), coronary artery bypass grafting or other coronary revascularisation, but not angina pectoris. Where World Health Organisation International Classification of Disease (ICD) codes were available, fatal CHD events included ICD-10 codes I-21, I-22, I-23, I-24 and I-25, and ICD-9 codes 410.0 to 410.9. Stroke of all aetiology (including haemorrhagic and ischaemic events)was included. Fatal stroke events included ICD-10 codes I-61 to I-64, I-67 and I-69, and ICD-9 codes 430.0 to 434.9.

Where data allowed, only the first recorded cardiovascular event (of any type) was included and all subsequent events excluded from the analysis. Prevalent events were defined as those occurring prior to study baseline and incident events as any event occurring during follow-up.

SNP selection

We aimed to compile the smallest set of variants to yield the most data on effects of *IL6R* genotype and therefore act as the most robust instruments for variation in the gene. A number of open-access populations of European ancestry were searched via the Genome Variation Server and the International HapMap Project (Release 23a/Phase 3)[51] for SNP within and 20kbp up- and downstream of *IL6R*. Using HaploView[52] and existing genotype data from Whitehall II we identified tagging SNPs present on the IBC HumanCVD BeadChip v2 (Cardiochip). SNPs were included on the HumanCVD BeadChip (Cardiochip) platform array - a candidate gene-based SNP array including a high density of markers in nearly 2,000 genes implicated in CVD - in part for their ability to tag genetic variation at this locus. Of those included on Cardiochip, we excluded SNPs whose associated p-value for univariate linear regression of circulating $\log_e$ IL-6 levels on genotype conducted using PLINK in Whitehall II participants was greater than $p=1x10^{-5}$ in an additive model. For each SNP we also assessed the beta-coefficient for the above linear regression model and the minor allele frequency in Whitehall II and the HapMap CEU samples. Using data from the 1000 Genomes Project Pilot 1, we evaluated linkage disequilibrium (LD) between each of the resulting shortlisted SNPs and between those and SNPs identified by previous genome-wide association studies of quantitative traits, giving preference to SNPs capturing data for previously identified SNPs or multiple IL-6-associated SNPs in Whitehall II. Where possible we excluded SNPs

in strong LD (r^2>0.9) to assemble the final subset of SNPs for genotyping across the collaborating studies. The consequence and function of each selected SNP was assessed using HapMap, NCBI SNP database (http://www.ncbi.gov.uk/snp), Ensembl (http://www.ensembl.org), UCSC Genome Browser[53].

Genotyping and genotype data quality control

Genotypes were coded as 0, 1 or 2 homozygous common allele, heterozygous and homozygous minor allele respectively.

We included existing genotype data where available and commissioned new genotyping where necessary.

Studies used a variety of genotyping platforms including: the ITMAT/Broad Institute CARe Consortium HumanCVD BeadChip ('Cardiochip')[54]; Illumina Metabochip, Illumina 550k v1/3 and 610/660K (Illumina, San Diego CA, USA); Affymetrix GeneChip Human Mapping 500K and Affymetrix Genome-Wide Human SNP Array 6.0 (Affymetrix, Santa Clara, CA, USA); the ABI TaqMan (Applied Biosystems, Foster City, CA, USA); or KASPar (KBiosciences, Hoddesdon, UK) [add other collaborator platforms].

Statistical analysis

Participant inclusion criteria

We restricted the analysis to unrelated individuals with available data for genotype at the lead SNP (or one of its proxies), and for at least one biomarker or clinical outcome.

Relatedness

Where study family structure was recorded, in each group of related participants all but the eldest of the group were excluded from the analyses.

SNP associations with continuous risk factors and circulating biomarkers

Analyses were based on an additive genetic model justified on the basis of the association of the three *IL6R* SNPs including rs7529229 (the most widely typed of these SNPs across all studies) with the circulating concentration of IL-6. Weighted mean difference between genotypes was calculated using the 'metan' command in Stata v 11.2, applying inverse-variance weighted fixed effects meta-analysis to the mean of each biomarker by genotype. Using the common homozygous group as a reference, the difference between the reference and

heterozygous and rare homozygous groups was estimated. Where biomarkers were $\log_e$ transformed, the proportional difference between geometric means was calculated. Linear regression models (using the 'regress' command in Stata and equivalent procedures in other analysis packages) were used to estimate differences (and 95% confidence intervals, CI) in biomarkers per additional minor allele possessed at each SNP. For biomarkers that had been $\log_e$ transformed, the beta-coefficient from such a regression model was exponentiated to find the proportional difference in geometric means associated with possession of each additional minor allele. Within study estimates of per-allele effects were combined using Mantel-Haenszel fixed effects meta-analysis.

Subgroup analyses

SNP associations with continuous biomarkers and with clinical outcomes were analysed with stratification by the following subgroups: males, females, individuals aged <55 years, 56-65 years and >65 years; individuals with and without CVD at the time of biomarker measurement; those with and without type-2 diabetes mellitus; users and non-users of lipid-lowering drugs; individuals with 10 year Framingham CHD risk of above and below 20%; individuals stratified by tertiles of non-HDL-cholesterol; body mass index <25kg/m^2, 25-30kg/m^2 and >30kg/m^2; genotyping platforms and study designs. Tests of interaction between subgroups were performed using the chi-squared test for heterogeneity within the 'metan' command in Stata.

SNP associations with clinical outcomes

We estimated for each disease endpoint the odds ratio (OR) and 95% CI per minor allele for each SNP (rs7529229, rs4845371, rs12740969) under an additive model, as above, where data were available. Per-allele odds ratios were calculated by logistic regression models (using the 'logit' command in Stata) and resulting odds ratios combined using inverse-variance weighted fixed effects meta-analysis to derive a summary estimate (the 'metan' command in Stata v11.1). These analyses included only studies where at least one case and one control for the relevant outcome were available. Sensitivity analysis using different genetic models (heterozygous vs. common allele homozygous, and minor allele homozygous vs. common allele homozygous) were also conducted (data available on request).

In studies where all participants were diabetic, individuals free of diabetes from EAS were used as controls for ET2DS and from Whitehall-II for EDSC and UDACS when calculating odds ratios by logistic regression with type 2 diabetes as the outcome.

Systematic review of treatment trials and other investigations of tocilizumab

Search strategy

We searched PubMed on 7 September 2010 using the keyword "tocilizumab" and supplemented this by a search of reference lists of existing reviews and a systematic review of tocilizumab treatment published by the Cochrane Collaboration[55].

Data extraction and analysis

Data were extracted by MVH and information extracted from a random selection of eligible studies was checked by DIS. CRP was selected as a biomarker by which the magnitude of tocilizumab's treatment effect could be indexed since it was the most widely reported at the greatest number of treatment doses and time-points for which data were available in the genetic studies. For continuous variables other than CRP, the difference from baseline in the active arm was compared to the difference in baseline of placebo for 8mg/kg vs. placebo RCT only. The most parsimonious time-points were chosen to maximise data yield across studies and also to prevent studies from being represented twice. For CRP, values were extracted for RCTs and cohort studies investigating 4mg/kg, 8mg/kg or 16mg/kg TCZ versus placebo at 8-12 weeks following initial treatment. For RCT, the difference in CRP from baseline in the intervention arm was compared to that of the difference in the placebo group. For cohort studies, only the difference between baseline and follow-up was compared (as there was no comparator arm). We abstracted data for all clinically relevant biomarkers and safety endpoints. Trial results were combined with Mantel-Haenszel (binary traits) and inverse variance (continuous traits) fixed effects meta-analysis performed using Stata 11.2 (StataCorp, Texas, USA).

Supplementary results

IL-6, CRP, fibrinogen and blood lipid concentrations

Four studies were conducted exclusively in men (BRHS, CaPS, HIFMECH, and NPHS-II) and two exclusively in women (BWHHS and WHI), all remaining studies contributed participants from both genders (with the proportion of men in the range 18% and 75%).

Study-specific geometric mean concentrations (approximate SD) were in the range 1.01 (0.66) to 3.49 (2.48)pg/ml for IL-6 (17 studies, 29,978 individuals), 0.85 (0.99) to 3.10 (3.49) mg/L for CRP (31 studies, 77,290 individuals) and 1.99 (0.44)g/L to 3.57 (0.77)g/L for fibrinogen (19 studies, 53,480 individuals). Total cholesterol concentration, available in 35 studies (n=119,565) was in the range (mean, SD) 3.71 (1.93) to 7.06 (1.33) mmol/L; LDL-C, available in 30 studies (n=102,107) was in the range 2.19 (1.24) to 4.93 (1.17) mmol/L; HDL-C, available in 32 studies (n=109,688) was in the range 0.84 (0.26) to 1.68 (0.52) mmol/L; non-HDL-C available in 30 studies (n=100,762) was in the range 3.02 (0.87) to 5.61 (1.30) mmol/L; and triglycerides, available in 32 studies (n=109,926) was in the range (geometric mean, approximate SD) 0.76 (0.38) to 4.71 (3.35) mmol/L (**Supplementary Figure 5**).

SNP selection, quality control & genotype

The Genome Variation Server identified 44 SNPs and the HapMap database 67 SNPs in the region of IL6R, of which 26 and 24 respectively passed the MAF threshold of 30%. Of the HapMap variants, 13 were designated tagging SNPs. Of the 42 SNPs in *IL6R* included on the CardioChip, 12 met the p-value threshold of $p<1x10^{-5}$ for association with circulating log_e IL-6 at phase 3 of the Whitehall II study. Strong linkage disequilibrium ($r^2>0.9$) was noted between one of the CardioChip SNPs and SNPs identified by GWAS studies for C-reactive protein[56] and soluble IL-6 receptor[57]. Evaluation of effect size (indicated by linear regression beta-coefficient and R^2 values) and linkage disequilibrium between SNPs around the *IL6R* gene (**Supplementary figure 3**) suggested an ultimate subset of 3 SNPs. Of these, 2 (rs7529229 and rs4845371) were located within IL6R itself and one (rs12740969) in *TDRD10*, 34kbp from *IL6R*. The latter SNP was selected because it captured information on variation at the 3' end of *IL6R*. Carriage of the minor allele at rs7529229 resulted in higher circulating levels of IL-6, whilst the minor alleles at rs4845371 and rs12740969 were associated with lower IL-6 levels.

Association of additional IL6R SNPs with inflammatory biomarkers

The associations of the other *IL6R* SNPs (rs4845371, rs12740969) with IL-6, CRP and fibrinogen were consistent with but opposite in direction to the findings for the rs7529229 variant (**Supplementary figure 8**).

Reporting of IL6R variants associated with safety endpoints.

The NHGRI catalogue of genome-wide association studies lists 8 GWA studies of colorectal cancer (searched 14 April 2011) and 12 GWA studies of prostate cancer (searched 18 April 2011) of which none reported variants in *IL6R* in either main or supplementary results. Of the 16 GWA studies of breast cancer (searched 15 April 2011), one study reported SNPs in *IL6R* though there was no association of those with cancer [58] (**Figure 4**).

Association of IL6R variants with CHD events

Estimates for the association of the *IL6R* variant with risk of CHD were consistent between the present *de novo* analysis and previously published case-control study data for the rs4537545 variant in LD (r^2=1.0 in the HapMap CEU sample) with rs7529229 [1] (**Figure 3**).

Supplementary Tables

1- Collaborating study characteristics

2 - Data availability for biomarkers & events across studies

3 - Clinical event definitions

4 - Published associations of *IL6R* variants with biomarkers and non-cardiovascular outcomes

5 - Genotyping platforms and QC metrics, genotype frequency across studies

6 - SNPs within 55kb of *IL6R* on the Cardiochip platform and their associations with log IL-6 in the Whitehall II study

7 - Safety endpoints in trials of tocilizumab therapy

8 - Associations of the functional *IL6R* rs8192284 variant with IL-6, CRP and fibrinogen concentrations in the Whitehall II study

Supplementary Table 1 - Collaborating study characteristics

Study name	Study design	Geographical location	Sampling frame	Participants included	Baseline year(s)	% Male	Age (Mean ,SD)
Present genetic analysis							
**AAA**	RCT	UK	General practice	2,359	1998-2001	29	61.9 (6.6)
**ARIC**	Cohort	USA	Community	9,588	1986	47	54.3 (2.7)
**BRHS**	Cohort	UK	General practice	3,945	1978-1980	100	68.7 (5.5)
**BWHHS**	Cohort	UK	General practice	3,445	1999-2001	0	69.4 (5.5)
**CARDIA**	Cohort	USA	Community	1,443	1984	47	25.6 (3.4)
**CaPS**	Cohort	UK	Community	1404	1984-1988	100	56.7 (4.4)
**CFS**	Cohort	USA	Community (family)	135	1995	59	53.9 (14.7)
**CHS**	Cohort	USA	Community	3,952	1989	45	70.4 (5.3)
**COGS**	Case-control	UK	Clinic	1905	n/a	47	n/a
**CORGI**	Case-control	UK	Population	1978	n/a	51	51.0 (5.9)*
**EAS**	Cohort	UK	General practices	917	1987	51	64.3 (5.6)
**EDS**	Cross-sectional	UK	Diabetes clinic	319	2001-2003	61	63.7 (13.7)
**ELSA**	Cohort	UK	HSE respondents	5,504	1998, 1999, 2001	46	61.0 (9.6)
**Ely**	Cohort	UK	General practice	1,601	1990-1992	47	61.1 (9.2)
**EPIC-N**	Nested C-Ct	UK	Community	1,690	1993-1997	50	60.1 (9.0)
**EPIC-NL**	Nested C-Ct	Netherlands	Existing cohorts	5,194	1993-1997	22	54.1 (10.1)
**ET2DS**	Cohort	UK	Community (cases)	1,069	2006-7	51	67.9 (4.2)
**FHS**	Cohort	USA	Community	1,336	1948/1971/2002	47	45.7 (10.1)
**HAPIEE-CZ**	Cohort	Czech Republic	Population	6,685	2002-2005	47	58.3 (7.1)
**HAPIEE-LT**	Cohort	Lithuania	Population	6,887	2002-2005	45	61.0 (7.6)
**HAPIEE-PL**	Cohort	Poland	Population	8,716	2002-2005	49	57.7 (7.0)
**HAPIEE-RU**	Cohort	Russia	Population	7,082	2002-2005	46	58.9 (7.1)
**HIFMECH**	Case-control	Europe	Cases/community	1,096	n/a	100	51.7 (5.5)
**IMPROVE**	Cohort	Europe	Clinic	3,465	2003	48	64.2 (5.4)
**InCHIANTI**	Cohort	Italy	Community	1,199	1998	44.8	68.4 (0.5)
**ISGS-SWISS**	Case-control	USA	Hospital/community	2,558	2001	52	66.1 (13.7)
**MedStar**	Cohort	USA	Clinic	866	2004-7	67	53.5 (10.2)
**MESA**	Cohort	USA	Community	2,298	2000	48	65.7 (10.2)
**MRC Fenland - GWAS†**	Cohort	UK	General practices	1,376	2004 (ongoing)	44	45.0 (7.3)
**MRC Fenland - Metabochip†**	Cohort	UK	General practices	3,059	2004 (ongoing)	47	47.0 (7.1)
**MRC NSHD**	Birth cohort	UK	Birth register	2,402	1946	53	53 (0)
**NPHS-II**	Cohort	UK	General practices	2,755	1989-1994	100	56.6 (3.4)
**PREVEND**	RCT	Netherlands	Community	3,867	1997	51	49.6 (12.5)
**PROSPER**	RCT	Europe	Clinic	5,244	1997-1999	48	75.3 (3.4)
**SMART**	Cohort	Netherlands	Clinic	8,026	1996	68	56.5 (12.4)
**TPT**	RCT	UK	General practice	5,085	1984-1989	100	57.4 (6.7)
**UCP**	Nested C-C	Netherlands	Clinic	1,632	2007	75	62.8 (9.6)
**UDACS**	Cross-sectional	UK	Diabetes clinic	585	2001-2002	59	66.7 (11.0)
**WHI**	Nested C-C	USA	Community	5,723	1994-1998	0	67.8 (6.7)
**Whitehall II**	Cohort	UK	Workplace	5,059	1985-1988	74	49.2 (6.0)
Published data							
**GerMIFS II**	Case-control	Germany	Clinic	2,520	n/a	NR	NR
**INTERHEART E**	Case-control	International	Clinic	1,691	n/a	NR	NR
**ISIS**	Case-control	International	Clinic	3,566	n/a	NR	NR

**LOLIPOP E**	Cohort	UK	Community	6,657	n/a	NR	NR
**PROCARDIS**	Case-control	UK	Clinic	8,328	n/a	NR	NR
**WTCCC**	Case-control	UK	Clinic	4,863	n/a	NR	NR

Footnote:
All cohort studies were prospective
The MRC Fenland Study is a single cohort though was genotyped using two platforms with different individuals in each group and therefore is reported as two distinct samples here.
C-C : case-control; C-Ct: case-cohort; HSE - Health Survey for England
*Mean age in controls

Supplementary Table 2 - Data availability for biomarkers & events in studies contributing to the primary genetic analysis

	Circulating biomarkers							Clinical events (prevalent & incident)		
	**IL-6**	**CRP**	**Fibrinogen**	**TC**	**HDL-C**	**LDL-C**	**Non-HDL-C**	**CHD events**	**Stroke**	**Diabetes**
**AAA**	-	1,926	2,064	2,260	-	-	-	95	48	48
**ARIC**	299	7,583	9,495	9,569	9,571	9,414	9,569	1,837	522	1,184
**BRHS**	2,261	3,863	3,865	3,853	3,742	3,761	3,739	668	424	289
**BWHHS**	3,257	3,162	3,258	3,257	3,253	3,177	3,253	655	252	458
**CARDIA**	226	1,315	681	1,435	1,435	1,435	1,435	10	4	99
**CaPS**	782	949	942	1,337	1,298	1,337	1,337	281	132	119
**CFS**	39	39	36	39	39	38	39	16	13	17
**CHS**	3,635	3,923	3,917	3,942	3,937	-	3,937	128	551	-
**EAS**	632	654	871	889	884	884	884	312	123	31
**EDS**	-	79	-	305	284	265	281	51	22	319
**ELSA**	-	5,433	5,401	5,436	5,434	5,292	5,434	-	-	-
**Ely**	-	624	-	1,600	1,598	1,597	1,598	-	-	-
**EPIC-N**	-	-	-	-	-	-	-	-	-	727
**EPIC-NL**	-	4,343	-	4,343	3,586	4,267	3,066	1,303	553	1,112
**ET2DS**	1,031	1,017	1,030	1,025	1,025		1,025	145	60	1,053
**FHS**	766	314	310	848	847	847	847	159	64	161
**HAPIEE-CZ**	-	6,486	-	6,546	6,522	6,278	6,521	435	291	351
**HAPIEE-LT**	-	915	-	6,872	6,754	6,689	6,754	376	394	394
**HAPIEE-PL**	-	900	-	8,751	8,750	8,615	8,750	737	206	657
**HAPIEE-RU**	-	1,019	-	7,083	7,081	7,079	7,081	648	462	255
**HIFMECH**	779	1,017	988	1,013	-	-	-	509	3	59
**IMPROVE**	-	3,463	-	3,459	3,459	3,398	3,459	301	63	858
**InCHIANTI**	1,199	-	-	-	-	-	-	-	-	-
**ISGS-SWISS**	-	-	-	-	-	-	-	-	1,070	-
**MedStar**	-	-	-	-	-	-	-	421	-	-
**MESA**	2,256	2,289	2,288	2,293	2,291	2,261	2,291	100	32	220
**MRC Fenland GWAS**	-	1,328	-	1,373	1,373	1,364	1,373	-	-	-
**MRC Fenland Metabochip**	-	-	-	3,052	3,052	3,027	3,052	-	-	-
**MRC NSHD**	-	-	-	2,236	2,082	2,072	2,082	-	-	-
**NPHS-II**	-	2,210	2,653	2,646	1,776	1,678	1,763	269	66	269
**PREVEND**	-	3,664	-	3,842	3,784	3,702	3,763	289	34	147
**PROSPER**	5,129	5,154	5,103	5,243	5,242	5,241	5,242	589	241	807

**SMART**	-	4,465	-	7,979	7,973	7,535	7,971	-	-	-
**TPT**	-	-	3,692	3,740	-	-	-	299	72	-
**UCP**	-	-	-	-	-	-	-	632	-	-
**UDACS**	574	570	-	582	582	565	582	60	39	585
**WHI**	4,472	4,495	4,458	4,743	4,728	4,661	4,728	2,396	1,076	281
**Whitehall II**	2,501	3,328	1,615	3,014	3,018	1,526	2,954	418	114	336
**Total**	**29,978**	**77,290**	**53,480**	**119,565**	**109,688**	**102,107**	**100,762**			

Abbreviations: TC - total cholesterol; HDL-C - high density lipoprotein cholesterol; LDL-C - low density lipoprotein cholesterol

Supplementary Table 3 - Clinical event definitions

	**Prevalent CVD events**			**All CVD events** (prevalent & incident)					**All CHD events** (prevalent & incident)					**All stroke** (all aetiology, prevalent & incident)					**Diabetes** (all aetiology, prevalent & incident)				
	Non-fatal			Non-fatal			Fatal		Non-fatal			Fatal		Non-fatal			Fatal		Non-fatal			Fatal	
	Self report	Medical records	Clinical/lab. measures	Self report	Medical records	Clinical/lab. measures	Death certificate	ICD coded	Self report	Medical records	Clinical/lab. measures	Death certificate	ICD coded	Self report	Medical records	Clinical/lab. /imaging measures	Death certificate	ICD coded	Self report	Medical records	Clinical/lab. measures	Death certificate	ICD coded
AAA	●		●		●		●	●		●		●	●		●		●	●		●		●	●
ARIC		●			●		●	●		●		●	●		●		●	●		●	●	●	●
BRHS		●			●		●	●		●		●	●		●		●	●		●			
BWHHS		●		●	●		●	●	●	●		●	●	●	●		●	●	●	●		-	-
CaPS	●	●		●	●		●	●	●	●		●	●	●	●		●	●	●	●	●	●	●
CARDIA		●			●		●			●		●			●		●			●		●	
CFS	●					●			●					●						●			
CHS	●			●					●					●					●				
EAS	●	●	●	●	●	●	●	●	●	●	●	●	●	●	●		●	●	●			●	●
EDS	-	-	-	●	●		-	-	●	●		-	-	●	●		-	-		●	●	-	-
ELSA	-	-	-	-	-	-	-	-	-	-	-	-	-	-	-	-	-	-	-	-	-	-	-
Ely	-	-	-	-	-	-	-	-	-	-	-	-	-	-	-	-	-	-	-	-	-	-	-
EPIC-N	-	-	-	-	-	-	-	-	-	-	-	-	-	-	-	-	-	-	●	●	●	●	●
EPIC-NL	-	-	-		●		●	●		●		●	●		●		●	●	●	●		●	●
ET2DS	●	●	●	●	●	●	-	-	●	●	●	-	-	●	●		-	-		●	●	-	-
FHS		●			●		●			●		●			●		●			●		●	
HAPIEE-CZ	●			●	●	●	●	●	●	●	●	●	●	●	●	●	●	●	●			●	●
HAPIEE-LT	●			●	●	●	●	●	●	●	●	●	●	●	●	●	●	●	●			●	●
HAPIEE-PL	●			●	●	●	●	●	●	●	●	●	●	●	●	●	●	●	●			●	●
HAPIEE-RU	●			●	●	●	●	●	●	●	●	●	●	●	●	●	●	●	●			●	●
HIFMECH		●	●	●	●		-	-	●	●		-	-	●			-	-	●			-	-
IMPROVE	n/a	n/a	n/a	n/a	n/a	n/a	n/a	n/a	n/a	n/a	n/a	n/a	n/a	n/a	n/a	n/a	n/a	n/a	n/a	n/a	n/a	n/a	n/a
InCHIANTI	-	-	-	-	-	-	-	-	-	-	-	-	-	-	-	-	-	-	-	-	-	-	-
ISGS-SWISS	●			●			-	-	●			-	-		●	●	●		-	-	-	-	-
MedStar	-	-	-	-	-	-	-	-		●	●	-	-	-	-	-	-	-	-	-	-	-	-
MESA	●			●					●					●					●				
MRC Fenland - GWAS	-	-	-	-	-	-	-	-	-	-	-	-	-	-	-	-	-	-	-	-	-	-	-
MRC Fenland -Metabochip	-	-	-	-	-	-	-	-	-	-	-	-	-	-	-	-	-	-	-	-	-	-	-
MRC NSHD	-	-	-	-	-	-	-	-	-	-	-	-	-	-	-	-	-	-	-	-	-	-	-
NPHS-II		●		●	●		●	●	●	●		●	●	●	●		●	●	●	●		●	●
PREVEND		●			●		●	●		●		●	●		●		●	●		●		●	●

PROSPER	-	•	•	-	•	•	•	•	-	•	•	•	•	-	•	•	•	•	-	•	•	•	•
SMART	-	-	-	-	-	-	-	-	-	-	-	-	-	-	-	-	-	-	-	-	-	-	-
TPT		•			•		•			•		•			•		•			•		•	
UCP	-	-	-	-	-	-	-	-		•				-	-	-	-	-	-	-	-	-	-
UDACS	-	-	-	•	•		-	-	•	•		-	-	•	•		-	-		•	•	-	-
WHI	•			•					•					•					•				
Whitehall II		•			•		-	-		•		-	-		•		-	-		•	•		

Supplementary Table 4 - Published associations of *IL6R* variants with biomarkers and non-cardiovascular outcomes

Endpoint	Source	GWAS	SNP (minor allele)	Cases/controls (studies)	LD with rs7529229 (r^2)[c]	Odds ratio (95% CI)	p-value
All cancer	Present studies	No	rs75299229 (T)	3,828/40,006(10)	-	0.98 (0.93 to 1.03)	0.39
	Published [59]	No	rs8192284 (C)	1,548/17,117 (1)	0.97	1.05 (0.95 to 1.16)	0.38
	Pooled estimate			**5,376/57,123 (11)**	-	**0.99 (0.95 to 1.04)**	**0.66**
Breast cancer	Present analysis	Yes	rs4537545 (A)	13,574/14,852 (1)	1.00	1.01 (0.94 to 1.10)	0.74
	Published [60]	Yes	rs4537545(A)	2,702/5,726 (1)	1.00	0.97 (0.90 to 1.05)	0.50[a]
	Pooled estimate			**14,456/15,758 (2)**	-	**1.03 (0.96 to 1.10)**	**0.40**
Respiratory infection	Published [61]	No	rs8192284 (C)	264/287	0.97		0.19[b]
Colorectal cancer	Present analysis	No	rs4537545 (A)	1863/1903 (2)	1.00	1.03 (0.96 to 1.12)	0.40

p-value thresholds for declaration of significance: [a] $1x10^{-6}$; [b] 0.05;

* Discovery dataset

†Replication dataset

[c] LD data from 1000 Genomes Pilot 1, assessed using SNP Association and Proxy Search tool (http:// http://www.broadinstitute.org/mpg/snap/, accessed 26 June 2011)

Supplementary Table 5 - Genotyping platforms and QC metrics, genotype frequency across studies

Study	Participants	Genotyping platform	Call rate (%)	Proxy SNP used (LD with rs7529229, r^2)*	TT (wildtype homozygotes)	TC	CC (variant homozygotes)	MAF (%)	Hardy-Weinberg χ^2	Hardy-Weinberg p=value
AAA	2,359	KASPar	96	-	771	1,128	369	41.1	1.65	0.199
ARIC	9,588	IBC Cardiochip	100	-	3,316	4,571	1,701	41.6	3.34	0.067
BRHS	3,945	KASPar	98	-	1,270	1,889	715	42.8	0.07	0.787
BWHHS	3,445	IBC Cardiochip	100	-	1,143	1,696	600	42.1	0.46	0.498
CaPS	1,404	KASPar	98	-	479	688	267	41.4	0.14	0.706
CARDIA	1,443	IBC Cardiochip	100	-	500	711	231	40.7	0.68	0.410
CFS	135	IBC Cardiochip	100	-	47	55	33	44.8	4.20	0.040
CHS	3,952	IBC Cardiochip	100	-	1,400	1,895	656	40.6	0.12	0.730
EAS	917	ABI TaqMan	97	-	314	405	171	42.0	3.85	0.050
EDS	319	ABI TaqMan	100	rs8192284 (0.97)	127	147	45	37.2[1]	0.06	0.814
ELSA	5,504	KASPar	99	-	1,807	2,646	1,015	42.8	0.72	0.397
Ely	1,601	Metabochip	100	rs4129267 (1.00)	536	765	300	42.6	0.86	0.35
EPIC-N	1,690	Metabochip	100	rs4129267 (1.00)	588	791	311	42.0	2.45	0.12
EPIC-NL	5,194	IBC Cardiochip	97	-	1,916	2,457	821	39.5	0.51	0.475
ET2DS	1,069	KASPar	99	-	369	512	172	40.6[1]	0.06	0.802
FHS	1,336	IBC Cardiochip	100	-	469	647	220	40.7	0.02	0.901
HAPIEE-CZ	6,700	KASPar	98	-	2,545	3,146	1,009	38.5	0.52	0.47
HAPIEE-LT	6,914	KASPar	99	-	3,106	3,073	735	32.9	0.38	0.54
HAPIEE-PL	8,758	KASPar	99	-	3,399	4,063	1,296	38.0	2.08	0.15
HAPIEE-RU	7,085	KASPar	98	-	3,176	3,111	798	33.2	0.75	0.39
HIFMECH	1,096	ABI TaqMan	97	-	381	477	204	41.7[2]	6.14	0.013
IMPROVE	3,465	Metabochip	n/a	rs7518199 (0.97)	1,377	1,621	467	36.9	0.09	0.77
InCHIANTI	1,199	Illumina 550k	>98	rs4129267 (1.00)	457	589	164	37.9	1.42	0.23
ISGS-SWISS	2,558	Illumina 500K v1/3 & 610/660K	>95	-	888	1,261	409	41.0	1.21	0.271
MedStar	851	Affymetrix 6.0	>95	-	321	412	118	38.1	0.61	0.44
MESA	2,298	IBC Cardiochip	100	-	743	1,143	412	42.8	0.58	0.447
MRC Fenland - GWAS	1,376	Affymetrix 500K	100	rs4537545 (1.00)	465	683	227	41.4	0.80	0.37
MRC Fenland - Metabochip	3,059	Metabochip	100	rs4129267 (1.00)	1,067	1,481	511	40.9	0.006	0.94
MRC NSHD	2,402	KASPar	100	-	806	1,139	457	42.7	2.33	0.127
NPHS-II	2,755	ABI TaqMan	97	-	940	1,219	508	41.9	10.00	0.002
PREVEND	3,876	KASPar	100	-	1,365	1,869	633	40.5	0.03	0.87

**PROSPER**	5,244	Illumina 660K	100	rs4537545 (1.00)	1,842	2,532	869	40.7	0.00	0.982
**SMART**	8,026	KASPar	>95	-	3,129	3,944	1,260	38.8	0.09	0.77
**TPT**	4,114	ABI TaqMan	92	-	1284	1852	638	41.4	0.46	0.50
**UCP**	1,632	IBC Cardiochip	100	-	583	788	261	40.1	0.04	0.85
**UDACS**	585	ABI TaqMan	100	rs8192284 (0.97)	127	147	45	37.2[1]	0.06	0.81
**WHI**	5,723	IBC Cardiochip	100	-	1931	2771	1010	41.9	0.09	0.770
**Whitehall II**	5,059	IBC Cardiochip	100	-	1710	2451	881	41.8	0.00	0.957

MAF - minor allele frequency

*Linkage disequilibrium (LD) data derived from 1000 Genomes Pilot 1

[1] Minor allele frequency in diabetes cases

[2] Minor allele frequency in CHD cases

Supplementary Table 6 - SNPs within 55kb of IL6R on the Cardiochip platform and their associations with log IL-6 in the Whitehall II study

				Association with log-IL-6			
**Selected for analysis**	**SNP**	**Chr.**	**Base pair position**	**No. of participants**	**β-coefficient (SE)**	**95% Confidence interval**	**Regression model p-value**
	rs7518199	1	152674043	4475	0.089 (0.012)	0.064 to 0.113	$1.52x10^{-12}$
	rs4129267	1	152692888	4473	0.088 (0.012)	0.064 to 0.113	$1.77\ x10^{-12}$
	rs4537545	1	152685503	4472	0.087 (0.012)	0.063 to 0.112	$2.73\ x10^{-12}$
✓	**rs7529229**	**1**	**152687402**	**4472**	**0.086 (0.012)**	**0.061 to 0.110**	**6.15** $x10^{-12}$
✓	**rs12740969**	**1**	**152753684**	**4479**	**-0.078 (0.013)**	**-0.103 to -0.052**	**2.20** $x10^{-09}$
	rs4553185	1	152677579	4469	-0.064 (0.012)	-0.088 to -0.039	$3.22\ x10^{-07}$
✓	**rs4845371**	**1**	**152674964**	**4462**	**-0.062 (0.013)**	**-0.087 to -0.038**	**6.78** $x10^{-07}$
	rs4509570	1	152703008	4476	-0.073 (0.015)	-0.102 to -0.044	$7.17\ x10^{-07}$
	rs4845625	1	152688691	4473	-0.062 (0.012)	-0.086 to -0.037	$7.92\ x10^{-07}$
	rs28638007	1	152687978	4485	-0.061 (0.012)	-0.086 to -0.037	$8.57\ x10^{-07}$
	rs7553796	1	152671030	4458	-0.061 (0.013)	-0.086 to -0.037	$9.64\ x10^{-07}$
	rs6667434	1	152675724	4476	-0.060 (0.013)	-0.085 to -0.036	$1.55\ x10^{-06}$
	rs4845618	1	152666639	4470	-0.055 (0.012)	-0.080 to -0.031	$9.26\ x10^{-06}$
	rs4845626	1	152690109	4486	-0.045 (0.016)	-0.078 to -0.013	0.006
	rs4845374	1	152693571	4484	-0.046 (0.017)	-0.079 to -0.013	0.006
	rs8192282	1	152668303	4488	-0.046 (0.017)	-0.079 to -0.013	0.007
	rs11804305	1	152691121	4486	-0.046 (0.017)	-0.079 to -0.013	0.007
	rs4329505	1	152699044	4481	-0.044 (0.017)	-0.077 to -0.011	0.009
	rs10159236	1	152698029	4486	-0.043 (0.017)	-0.076 to -0.010	0.010
	rs12083537	1	152647727	4475	0.035 (0.015)	0.005 to 0.065	0.021
	rs7411976	1	152649067	4481	0.103 (0.047)	0.010 to 0.196	0.029
	rs1386821	1	152648673	4480	0.028 (0.016)	-0.003 to 0.059	0.073
	rs2229237	1	152668420	4489	-0.068 (0.066)	-0.197 to 0.060	0.298
	rs1552481	1	152642977	4488	0.285 (0.338)	-0.378 to 0.948	0.399
	rs6427641	1	152647110	4479	0.007 (0.012)	-0.017 to 0.032	0.572
	rs12089132	1	152644960	4489	0.284 (0.586)	-0.865 to 1.432	0.628
	rs3887104	1	152643295	4477	-0.008 (0.016)	-0.040 to 0.024	0.640
	rs4075015	1	152655820	4465	-0.006 (0.013)	-0.031 to 0.019	0.643
	rs12096944	1	152663782	4487	0.104 (0.239)	-0.365 to 0.573	0.663
	rs4601580	1	152661041	4481	-0.003 (0.012)	-0.027 to 0.022	0.819
	rs12739228	1	152692814	4489	-0.007 (0.033)	-0.072 to 0.058	0.838
	rs1552482	1	152642862	4489	0.062 (0.338)	-0.601 to 0.726	0.853
	rs11265610	1	152647408	4487	0.062 (0.338)	-0.601 to 0.725	0.854
	rs28730735	1	152693656	4486	-0.016 (0.115)	-0.242 to 0.210	0.892
	rs4845617	1	152644522	4452	-0.001 (0.013)	-0.026 to 0.024	0.948
	rs2054855	1	152637562	4478	0.001 (0.018)	-0.035 to 0.037	0.957
	rs34099703	1	152668336	4489	-0.026 (0.586)	-1.175 to 1.122	0.964
	rs4845616*	1	152644193	4488	-	-	-

rs4845615*	1	152641901	4489	-	-	-
rs28730736*	1	152693674	4487	-	-	-
rs7537306*	1	152667629	4489	-	-	-
rs28730732*	1	152668229	4463	-	-	-

SNPs are ranked on p-value and β-coefficient; β-coefficients are reported on the log scale.

*There was no variation in genotype at these very rare SNPs in the Whitehall II study sample and consequently a regression model could not be fitted.

Supplementary Table 7 - Safety endpoints in trials of tocilizumab therapy

Endpoint(s)*	No. of RCTs	No. randomised	No. of events	Odds ratio (95%CI)†	P	I^2 (%)
**CARDIAC**						
Myocardial infarction	1	335	1	0.30 (0.01, 7.49)	0.47	-
Cardiac	1	410	2	0.99 (0.06, 15.94)	0.99	-
Serious cardiac	2	1629	6	1.31 (0.23, 7.42)	0.76	0
Hypertension	2	1629	70	1.41 (0.83, 2.39)	0.20	18.5
**VASCULAR**						
Vascular	3	1965	120	1.34 (0.90, 1.99)	0.15	0
**DEATH**						
Death	2	2346	8	1.86 (0.45, 7.60)	0.39	63.8
**NERVOUS SYSTEM**						
Nervous system	3	1964	245	1.26 (0.95, 1.68)	0.10	0
Serious nervous system	2	1629	10	1.40 (0.35, 5.53)	0.63	0
**RESPIRATORY**						
Serious respiratory	2	744	43	0.96 (0.51, 1.79)	0.89	0
**INFECTION**						
Infection and infestation	3	1964	705	1.30 (1.07, 1.58)	0.008	0
Upper respiratory tract infection	2	534	37	1.20 (0.61, 2.34)	0.60	0
Pneumonia	2	2019	9	0.87 (0.23, 3.29)	0.84	0
Serious infection	5	2854	70	2.29 (1.33, 3.92)	0.003	12.2
**IMMUNOSUPPRESSION**						
Grade 3 neutropenia (0.5 to <1.0 × 10^9/L)	3	2346	51	26.00 (5.02, 134.70)	9.34×10^{-6}	0
**NEOPLASIA**						
Neoplasia	2	1200	4	0.42 (0.06, 2.88)	0.38	0
**LIPIDS**						
Total cholesterol >6.2mmol/L or >240mg/dL	3	2420	391	4.87 (3.64, 6.51)	1.74×10^{-26}	0
HDL-C >+30% or >60mg/dL or 1.6mmol/L	4	2755	319	2.46 (1.88, 3.22)	5.06×10^{-11}	67.7
LDL >160mg/dL or >4.1mmol/L	3	2346	255	5.11 (3.52, 7.41)	7.18×10^{-18}	0
LDL:HDL lipoprotein index >30%↑	2	1555	266	1.97 (1.46, 2.67)	9.34×10^{-6}	0
Triglycerides >500mg/dL or >5.7mmol/L	1	1220	15	1.42 (0.45, 4.50)	0.55	-
ApoB:ApoA1 atherogenic index >30%↑	2	1555	150	1.26 (0.88, 1.81)	0.21	0
**HEPATIC ENZYMES**						
ALT >3x ULN	4	2755	94	6.96 (3.58, 13.50)	9.95×10^{-9}	0
AST >3u ULN	3	2420	30	4.75 (1.66, 13.62)	0.004	0

* **Endpoint(s) definitions. Cardiac**: Smolen[62] - not defined. **Serious cardiac**: Smolen – not defined; Genovese[63] – not defined. **Vascular**: Emery[64] – not defined; Smolen – not defined; Genovese – not defined. **Nervous system**: Genovese – not defined; Smolen – not defined; Emery – not defined. Serious nervous system: Genovese – not defined; Smolen – not defined. **Infection and infestation**: Genovese – not formally defined, including upper respiratory tract infections, skin and subcutaneous tissue infections; Emery – not defined; Smolen – not defined**.** **Serious infection**: Maini – not formally defined, including arthritis and 2 cases of sepsis; Kremer [65]- not formally defined, including lower respiratory tract and gastrointestinal infections; Genovese - not formally defined, including staphylococcal cellulitis, acute pyelonephritis, sepsis and pneumonia; Smolen – not formally defined, including urinary tract infection, pneumonia, cellulitis, peridiverticular abscess and upper respiratory tract infection; Emery - not formally defined, including staphylococcal polyarthritis, cellulitis, necrotising pneumonia, urosepsis, osteomyelitis. **Serious respiratory**: Smolen – not formally defined, including idiopathic pulmonary fibrosis; Emery – not defined. **Neoplasia**: Smolen – not defined; Kremer: solid malignancy in TCZ group (uterine cancer, n=1), solid malignancy in placebo group (breast cancer n=1).

† Pooled using Mantel-Haenszel fixed effects models

Supplementary Table 8 - Associations of the functional *IL6R* rs8192284 variant with IL-6, CRP and fibrinogen concentrations in the Whitehall II study

		**Whitehall II**
**Biomarker**	*n*	*% difference in geometric mean per minor allele (95% CI)*
IL-6	4,480	9.26 (6.62 to 11.96)
CRP	4,503	-9.35 (-13.66 to -4.83)
Fibrinogen	4,466	-0.87 (-1.79 to 0.06)

Supplementary Figures

1 - Mechanisms of IL-6 receptor production, IL-6 signalling and inhibition of the IL-6 receptor by tocilizumab

2 - Linkage disequlibrium and disease/biomarker associated SNPs in and around *IL6R* (1q21.3)

3 - Identification of publications contributing to the systematic review of trials of tocilizumab treatment

4 - Mean difference in CRP at 4 and 8mg/kg doses of tocilizumab at 4, 8 and 16 weeks of therapy

5 - Box and whisker plots illustrating summary values for the main inflammation and lipid markers evaluated

6 - Stratified associations of *IL6R* rs7529229 variant on (a) IL-6, (b) CRP, and (c) fibrinogen concentrations

7 - Association of *IL6R* rs7529229 with log CRP levels stratified by genotyping platform used in each study contributing to the analysis

8 - Association of the *IL6R* SNPs a. rs4845371 and b. rs12740969 with IL-6, CRP and fibrinogen concentration

9 - Stratified associations of *IL6R* rs7529229 with fatal and non-fatal coronary heart disease

10 - Association of *IL6R* rs7529229 with risk of CHD (fatal & non-fatal) stratified by genotyping platform used in each study contributing to the analysis

Supplementary Figure 1 - Mechanisms of IL-6 receptor production, IL-6 signalling and inhibition of the IL-6 receptor by tocilizumab

a. Production of soluble IL-6 receptor (sIL-6R) is influenced by Asp358Ala (rs8192284, tagged by rs7529229) variant in *IL6R*. Proteolytic cleavage of the mIL-6R is increased with carriage of the Ala allele resulting in greater soluble concentration of the soluble receptor and reduced numbers of functioning mIL-6R. Both classical (through the membrane-bound IL-6R) and trans-signalling (through the soluble IL-6R) pathways are active with either allele, however the preferential production of sIL-6R with the Ala allele results in increased trans-signalling and reduced classical signalling. The phenotypic effects associated with the Ala allele (and the minor allele at rs7529229) are consequences of this alteration in receptor production.

b. Tocilizumab blocks both the mIL-6R and the sIL-6R, inhibiting IL-6 signalling via both classical and trans-signalling pathways.

c. Comparison between the directions of effects of the *IL6R* variant and tocilizumab reflect the similarities and differences in the biological processes of the two scenarios.

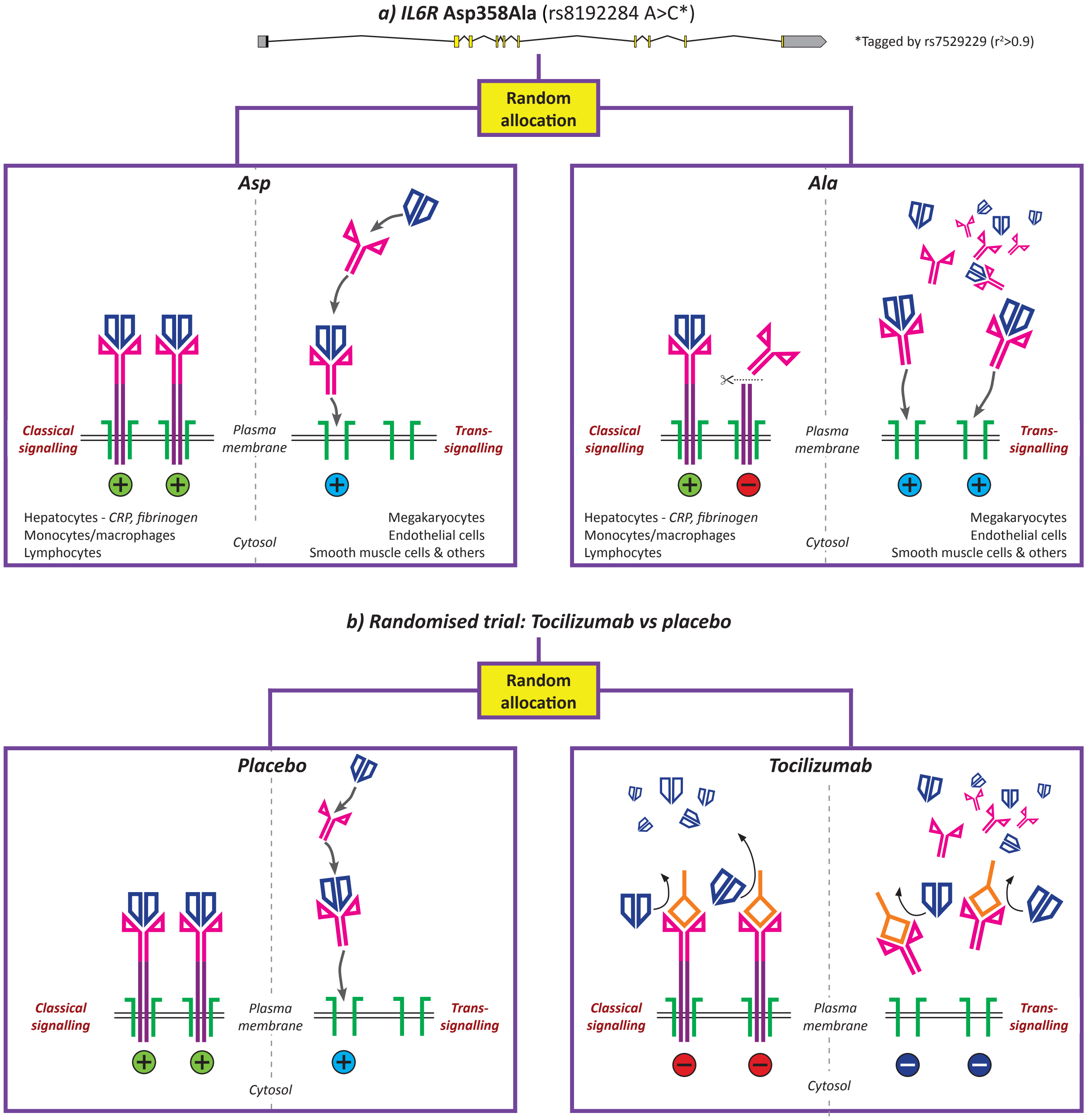

c) *IL6R* Asp358Ala vs Tocilizumab

	IL6R Asp vs Ala	Tocilizumab vs placebo	
sIL-6r production (cleavage)	↑	Not reported	
mIL-6r signalling	↓	↓	Upstream of IL-6R
sIL-6r signalling	↑	↑	Upstream of IL-6R
Plasma sIL-6r	↑	↑	Upstream of IL-6R
Plasma IL-6	↑	↑	Upstream of IL-6R
Plasma CRP	↓	↓	Downstream of IL-6R
Plasma fibrinogen	↓	↓	Downstream of IL-6R
CHD risk	↓	Not reported	Downstream of IL-6R

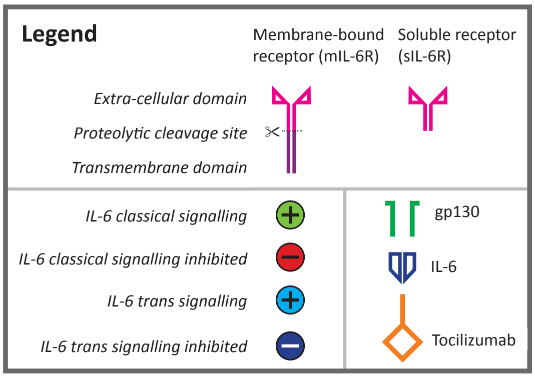

Supplementary Figure 2 - Linkage disequlibrium and disease/biomarker associated SNPs in and around *IL6R* (ch1q21.3)

a. (left panel) SNPs on chromosome 1q21.3 in the vicinity of IL6R based on HapMap Phase 3 build 36.

b. (centre panel) Prior disease and biomarker associations

c. (right panel) Linkage disequilibrium (r2) for SNPs contributing to the current analysis

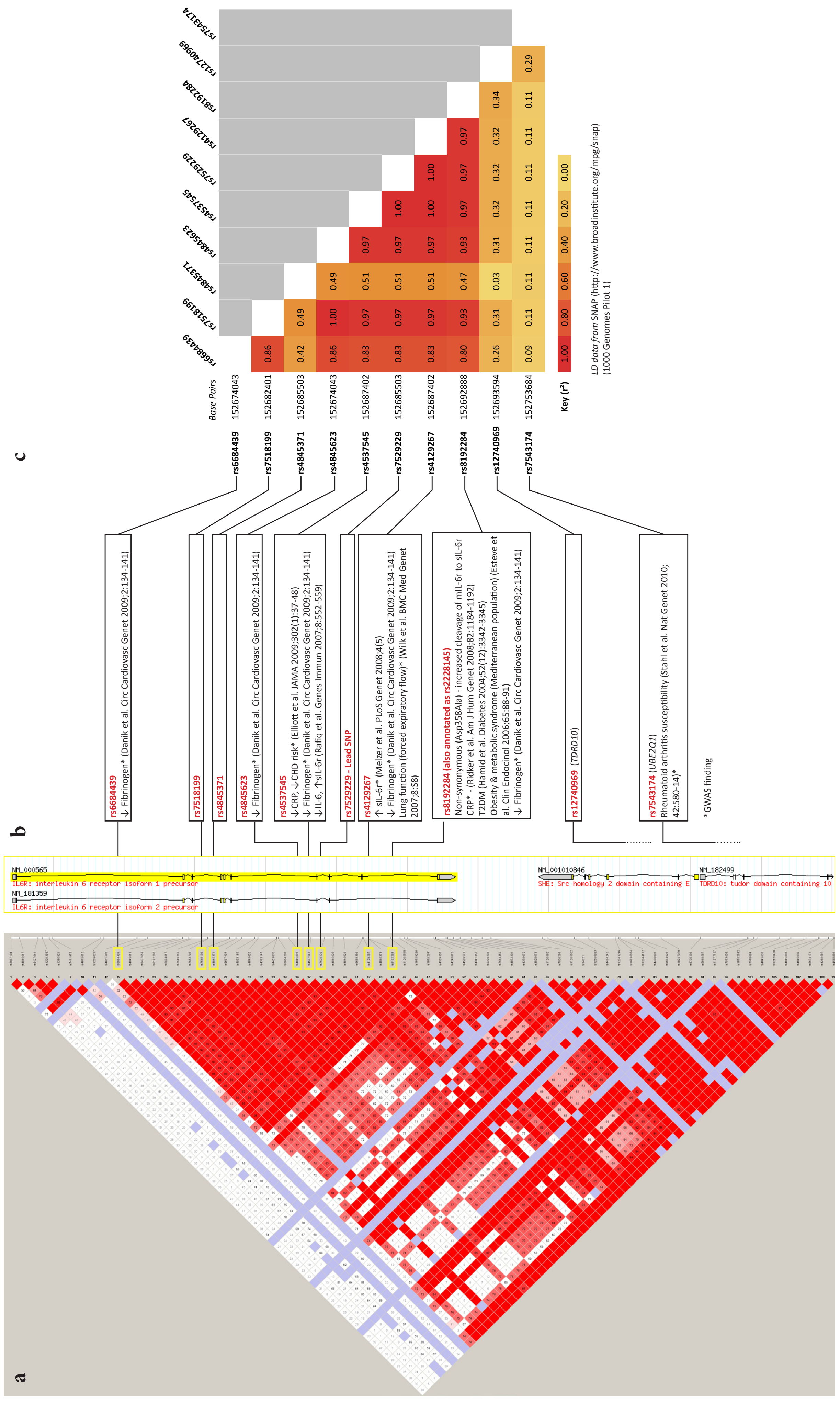

Supplementary Figure 3 - Identification of publications contributing to the systematic review of trials of tocilizumab treatment

PRISMA 2009 Flow Diagram

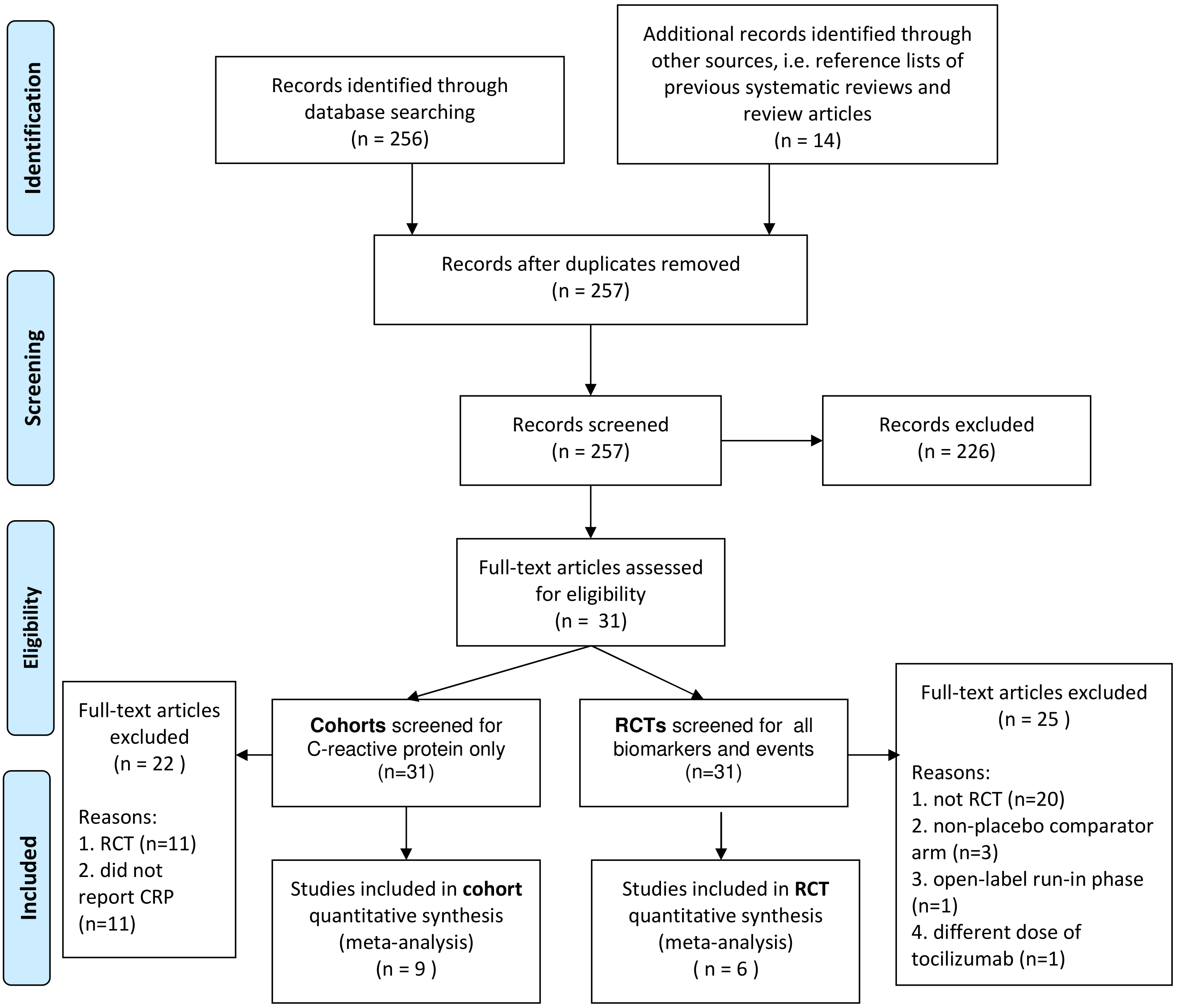

From: Moher D, Liberati A, Tetzlaff J, Altman DG, The PRISMA Group (2009). *P*referred *R*eporting *I*tems for *S*ystematic Reviews and *M*eta-*A*nalyses: The PRISMA Statement. PLoS Med 6(6): e1000097. doi:10.1371/journal.pmed1000097

For more information, visit www.prisma-statement.org.

Supplementary Figure 4 - Mean difference in CRP at 4, 8 and 16 mg/kg doses of tocilizumab at 4-12 weeks of therapy
Data pooled from randomised trials of tocilizumab versus placebo in patients with rheumatoid arthritis and observational studies of tocilizumab therapy.

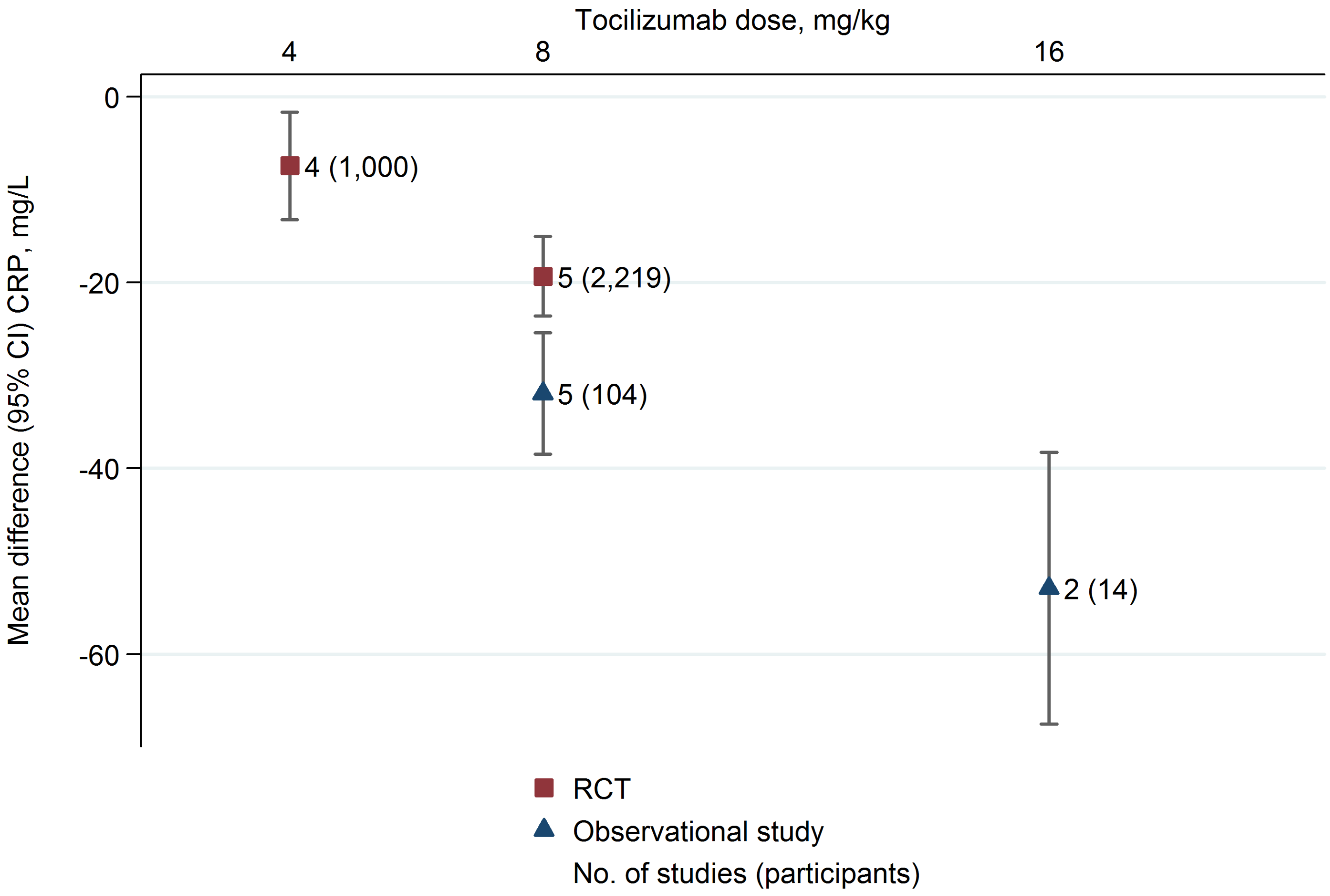

Supplementary Figure 5 - Box and whisker plots illustrating summary values for the main inflammation and lipid markers evaluated.

Plots show median (horizontal bar), interquartile range (box) and minimum/maximum (whiskers).

a

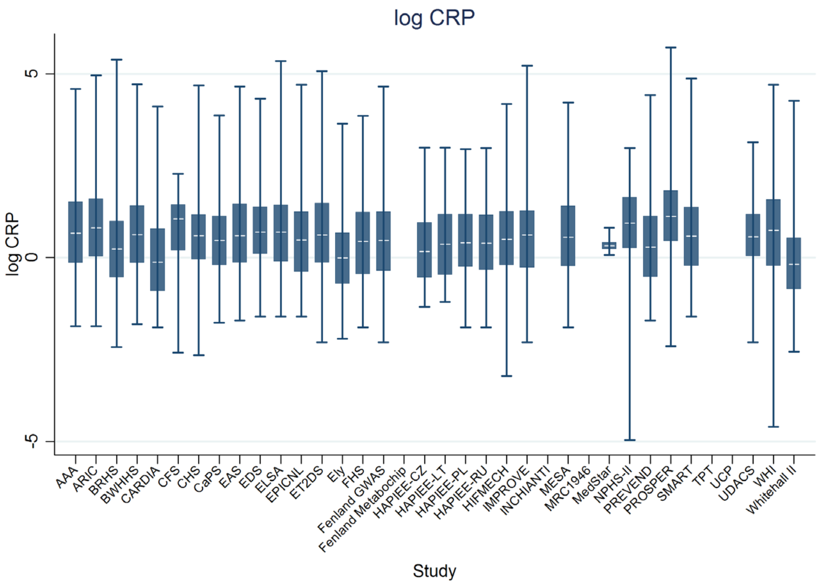

b

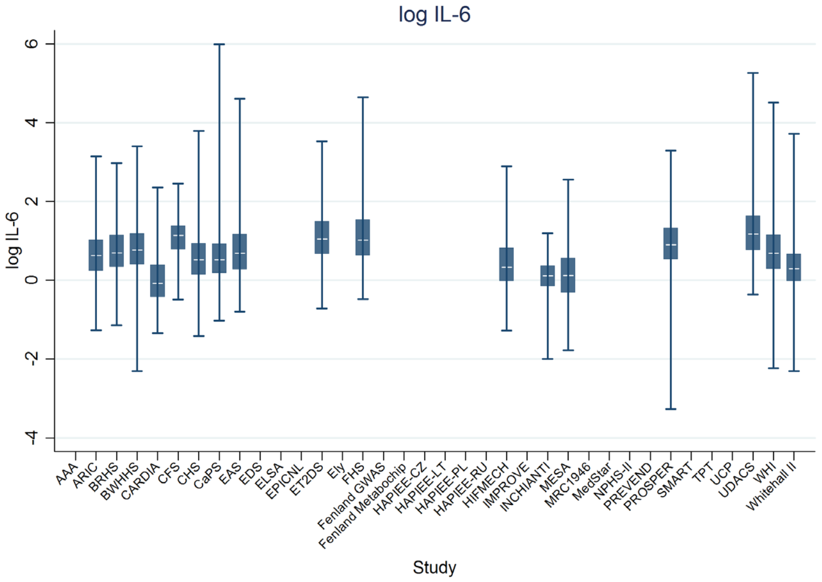

c

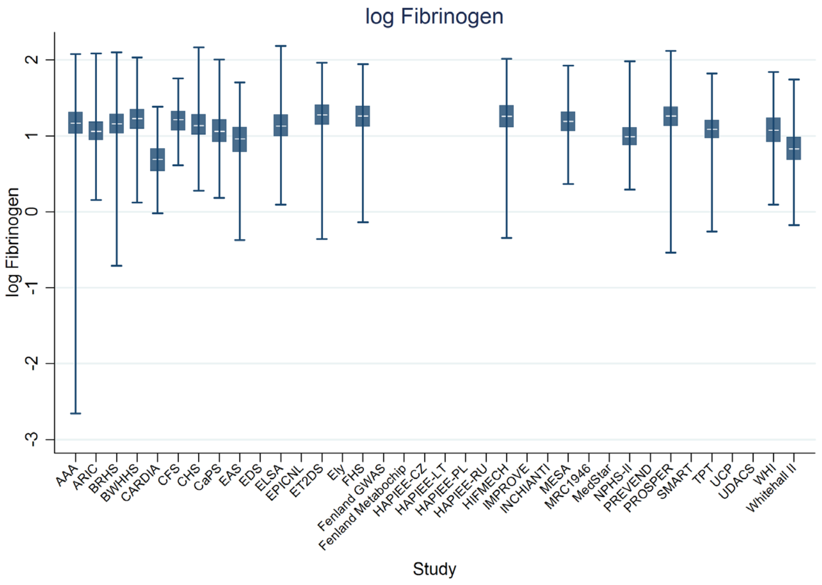

Supplementary Figure 5 continued

d

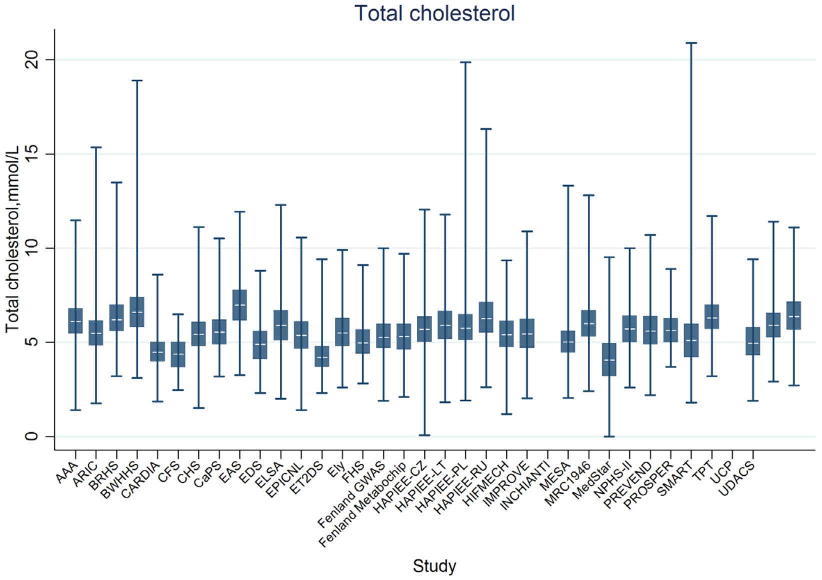

e

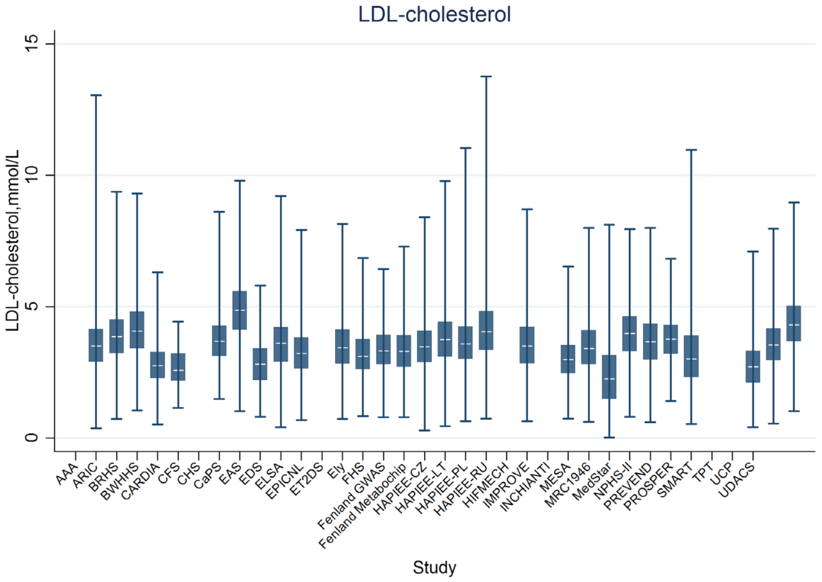

f

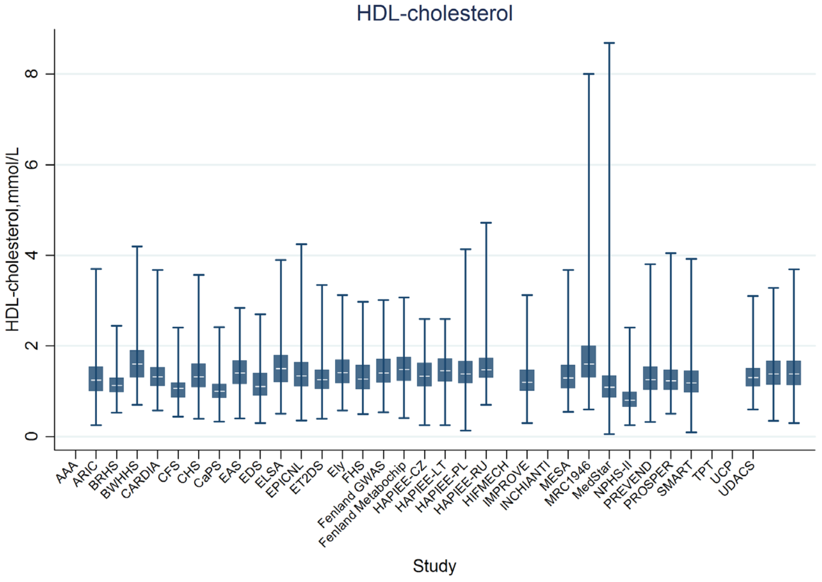

Supplementary Figure 5 continued

g

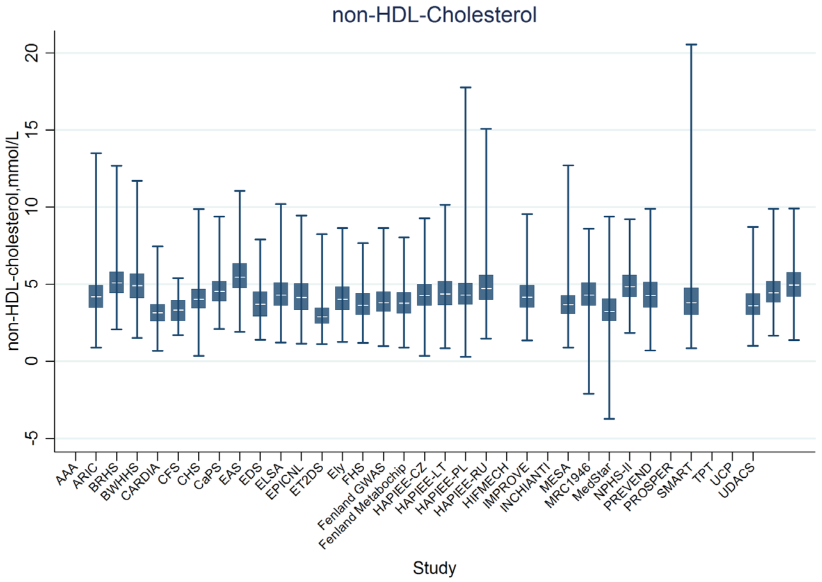

h

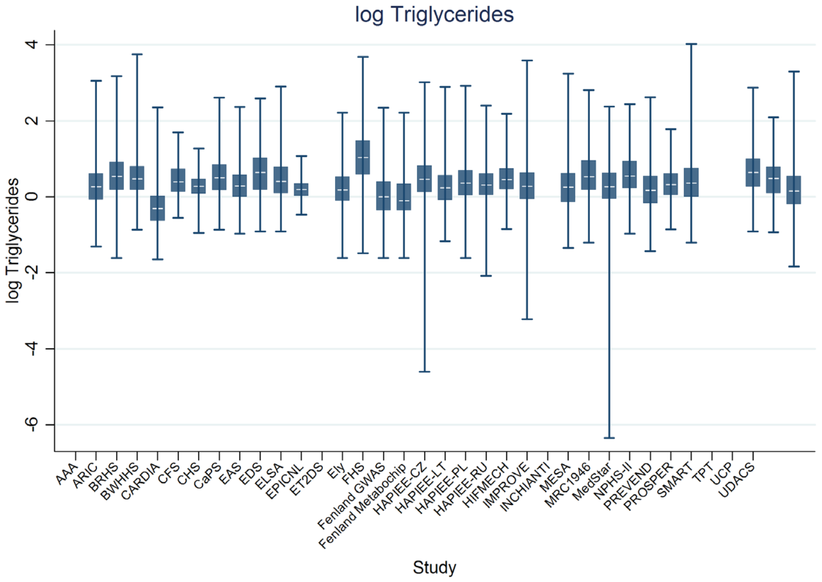

i

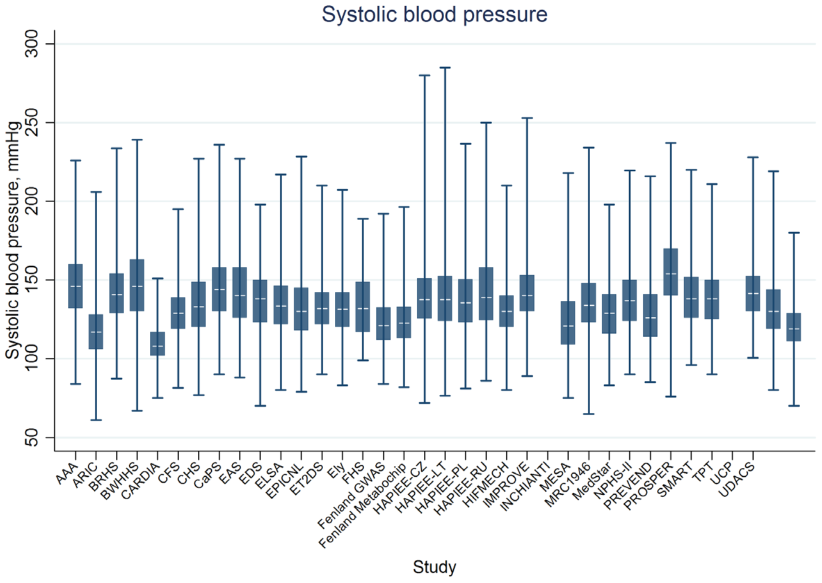

Supplementary Figure 5 continued

i

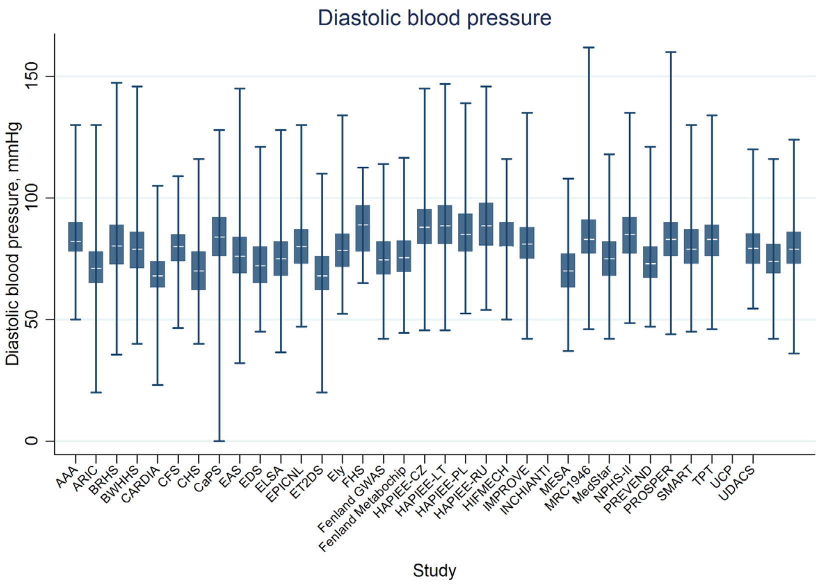

Supplementary Figure 6 - Stratified associations of *IL6R* rs7529229 variant on (a) IL-6, (b) CRP, and (c) fibrinogen concentrations.
Associations are stratified by age, prevalent CHD or diabetes status, Framingham score and non-HDL levels.

a

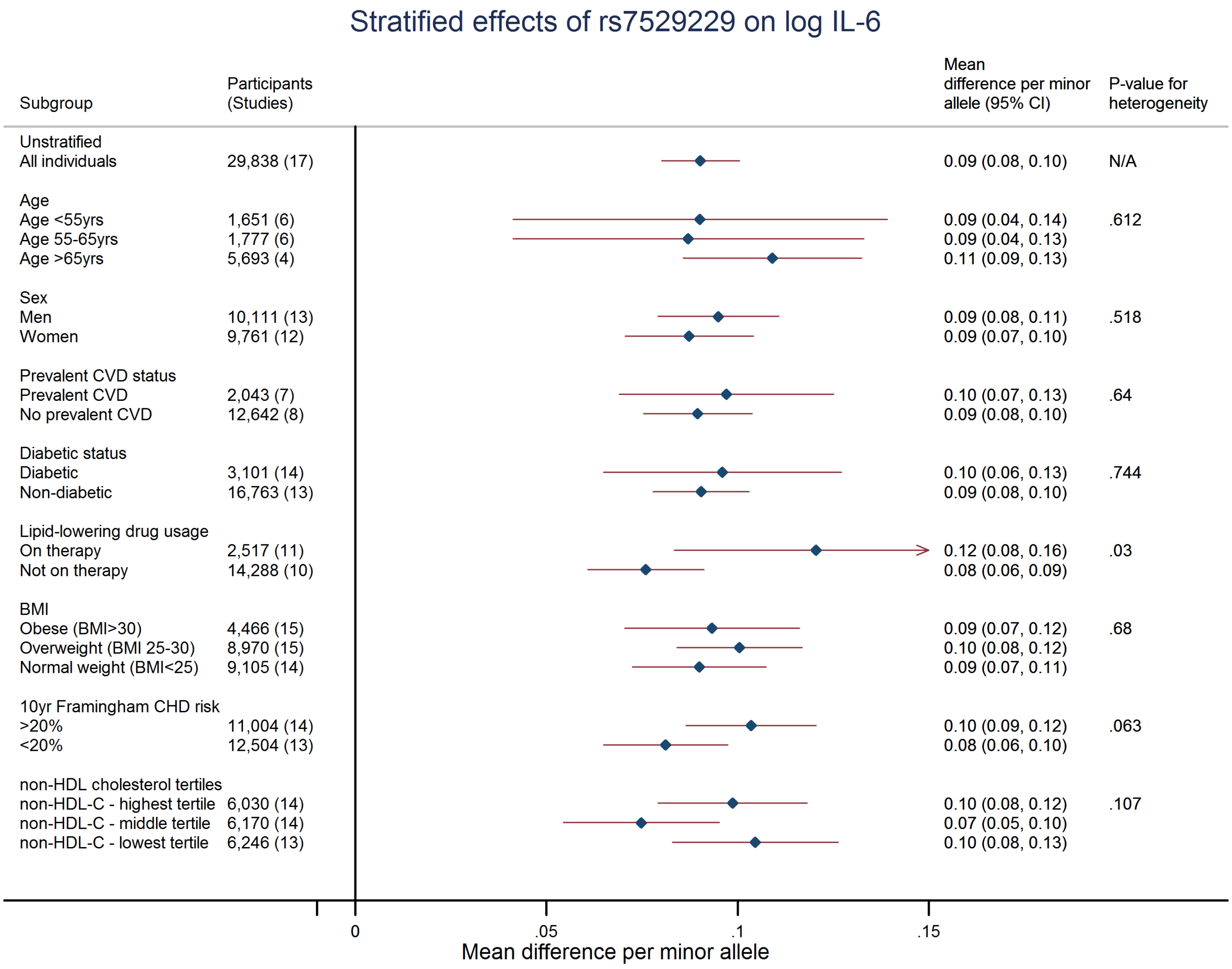

b

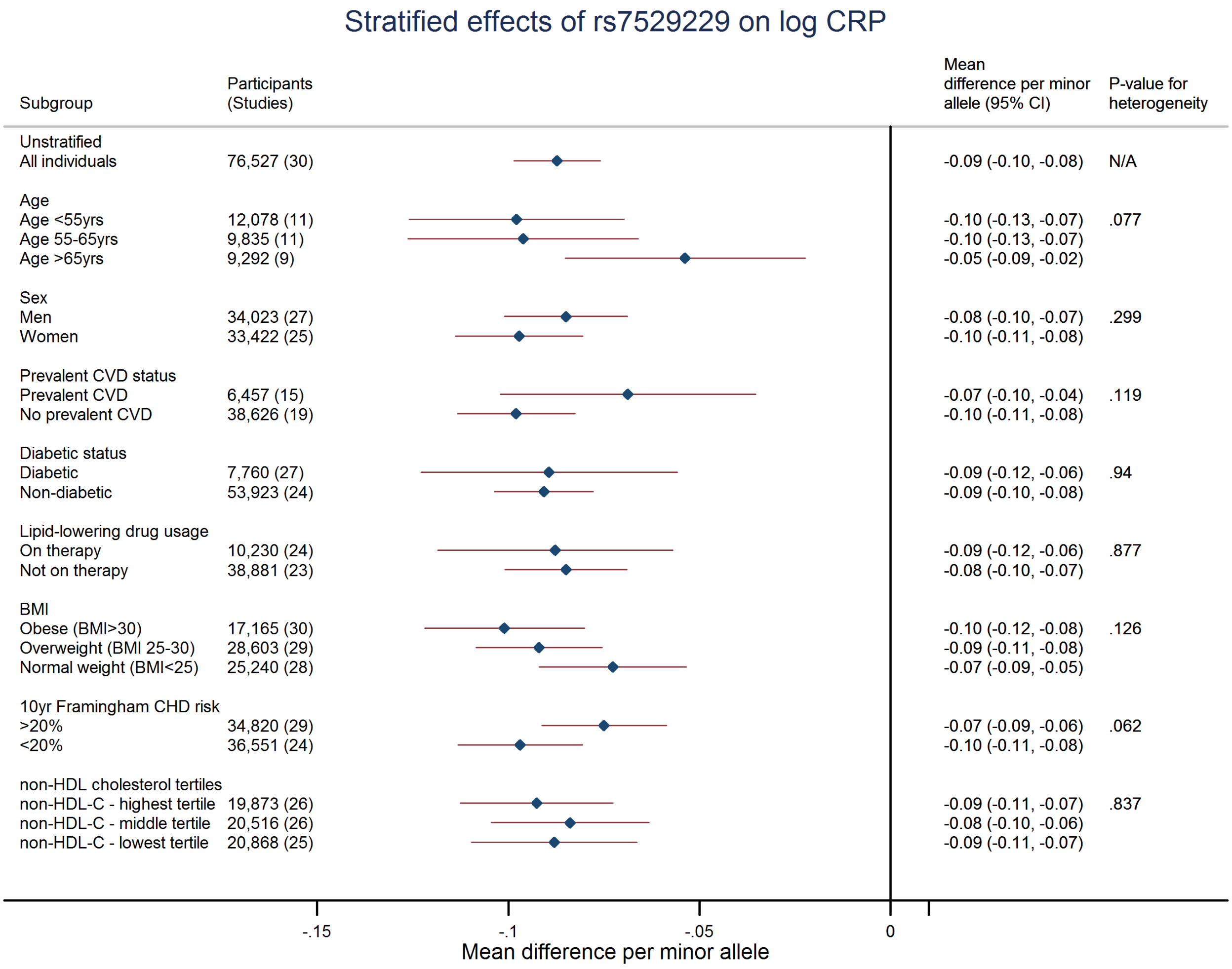
Stratified effects of rs7529229 on log CRP
Subgroup
Participants (Studies)
Mean difference per minor allele (95% CI)
P-value for heterogeneity
Unstratified
All individuals 76,527 (30) -0.09 (-0.10, -0.08) N/A
Age
Age <55yrs 12,078 (11) -0.10 (-0.13, -0.07) .077
Age 55-65yrs 9,835 (11) -0.10 (-0.13, -0.07)
Age >65yrs 9,292 (9) -0.05 (-0.09, -0.02)
Sex
Men 34,023 (27) -0.08 (-0.10, -0.07) .299
Women 33,422 (25) -0.10 (-0.11, -0.08)
Prevalent CVD status
Prevalent CVD 6,457 (15) -0.07 (-0.10, -0.04) .119
No prevalent CVD 38,626 (19) -0.10 (-0.11, -0.08)
Diabetic status
Diabetic 7,760 (27) -0.09 (-0.12, -0.06) .94
Non-diabetic 53,923 (24) -0.09 (-0.10, -0.08)
Lipid-lowering drug usage
On therapy 10,230 (24) -0.09 (-0.12, -0.06) .877
Not on therapy 38,881 (23) -0.08 (-0.10, -0.07)
BMI
Obese (BMI>30) 17,165 (30) -0.10 (-0.12, -0.08) .126
Overweight (BMI 25-30) 28,603 (29) -0.09 (-0.11, -0.08)
Normal weight (BMI<25) 25,240 (28) -0.07 (-0.09, -0.05)
10yr Framingham CHD risk
>20% 34,820 (29) -0.07 (-0.09, -0.06) .062
<20% 36,551 (24) -0.10 (-0.11, -0.08)
non-HDL cholesterol tertiles
non-HDL-C - highest tertile 19,873 (26) -0.09 (-0.11, -0.07) .837
non-HDL-C - middle tertile 20,516 (26) -0.08 (-0.10, -0.06)
non-HDL-C - lowest tertile 20,868 (25) -0.09 (-0.11, -0.07)
-.15
-.1
-.05
0
Mean difference per minor allele

Supplementary Figure 6 continued

c

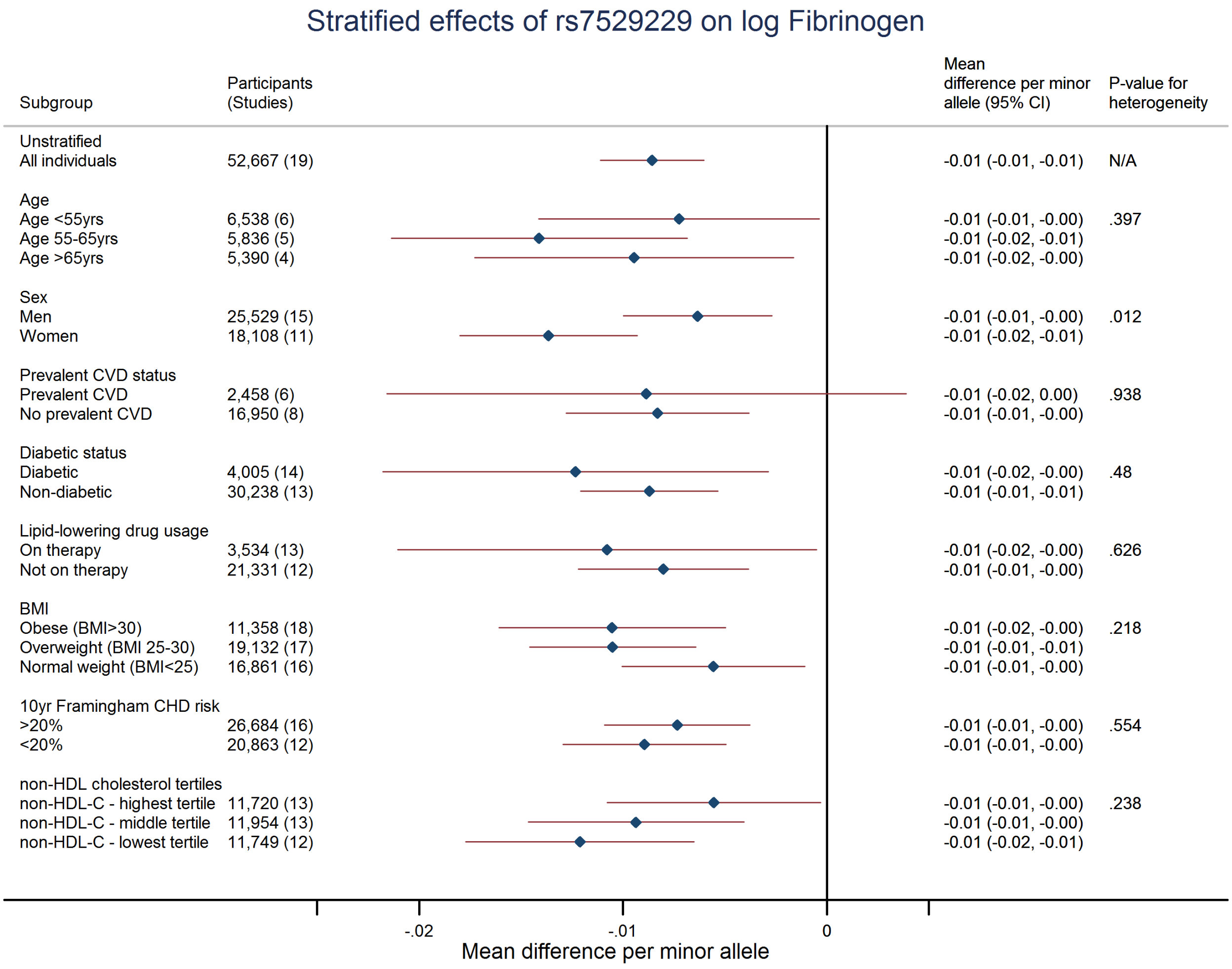

Supplementary Figure 7 - Association of *IL6R* rs7529229 with log CRP levels stratified by genotyping platform used in each study contributing to the analysis

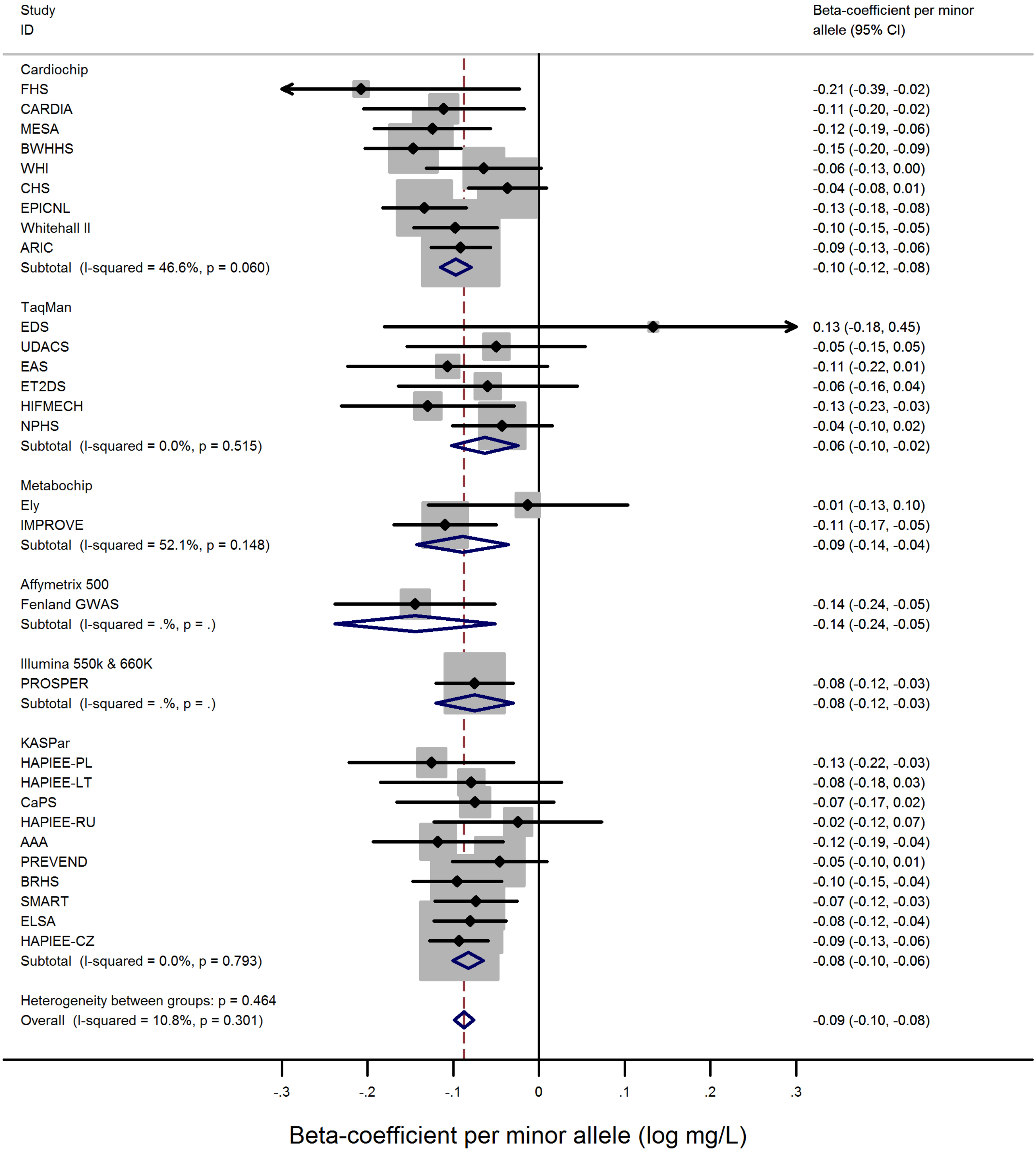

Supplementary Figure 8 - Association of the *IL6R* SNPs a. rs4845371 and b. rs12740969 with IL-6, CRP and fibrinogen concentration.

Estimates are based on pairwise comparison of individuals heterozygous or homozygous for the variant allele with reference to the wildtype homozygous group. The total number of participants (studies) is also shown.

a

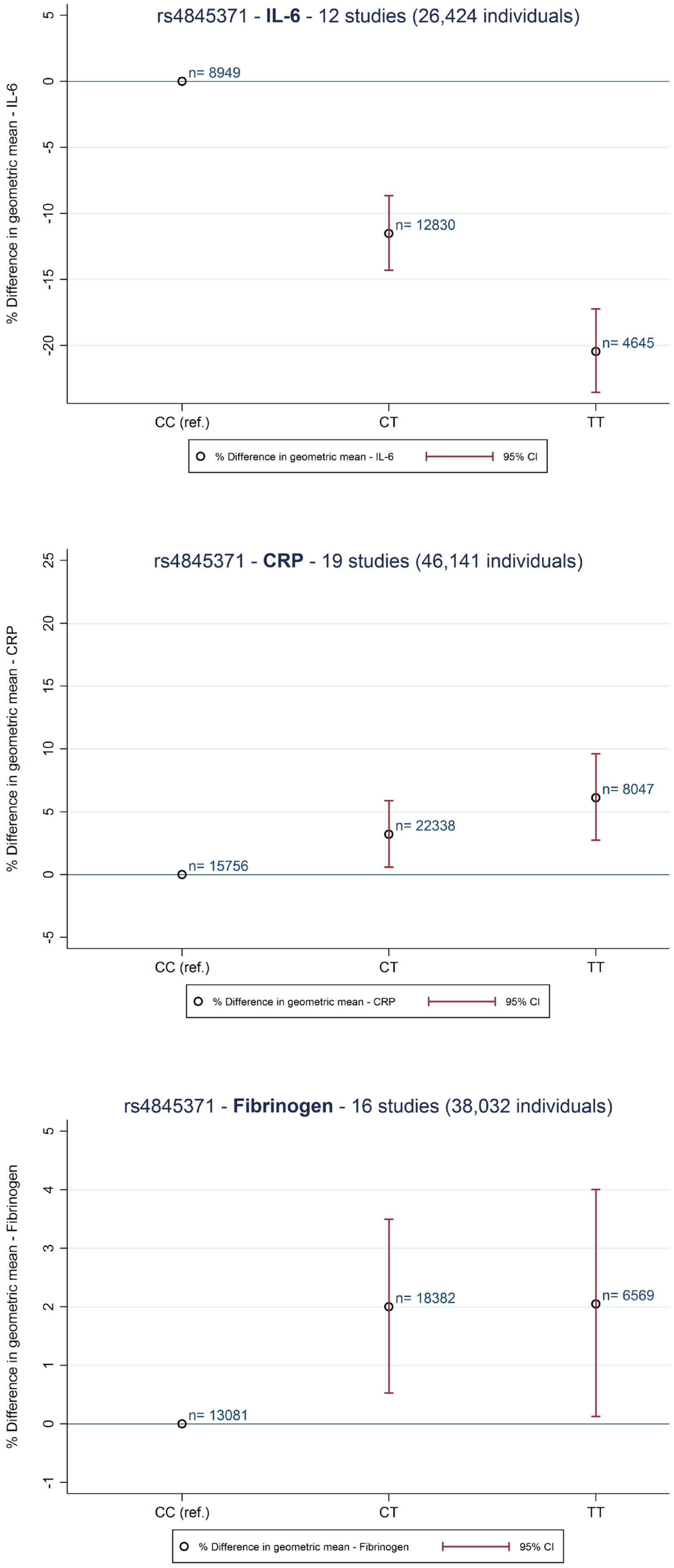

Supplementary Figure 8 continued

b

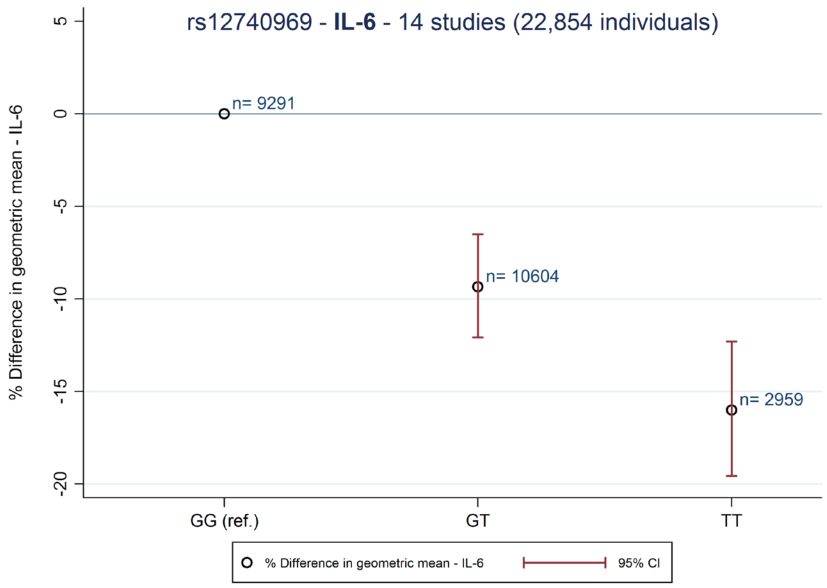

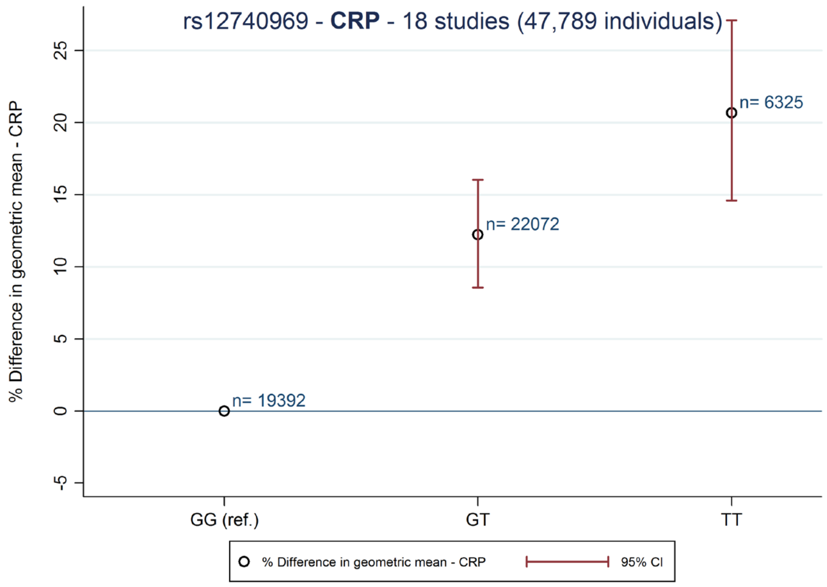

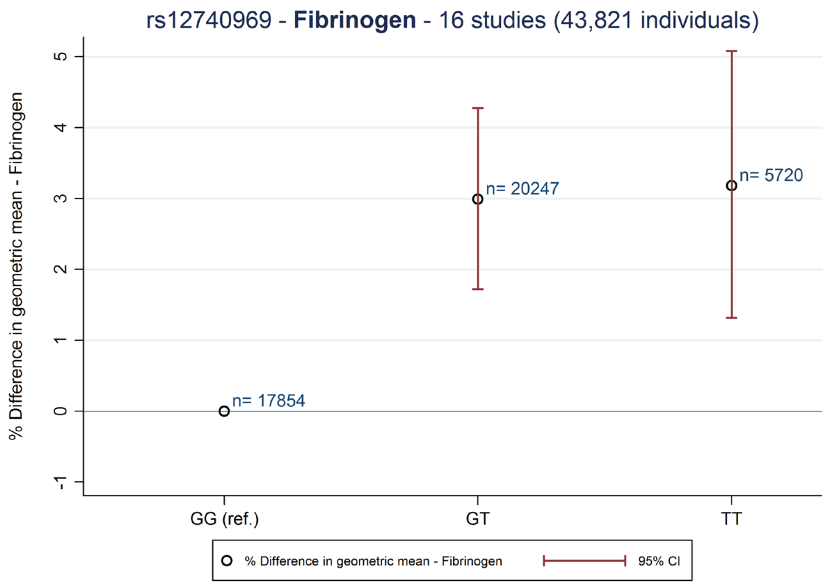

Supplementary Figure 9 - Stratified associations of *IL6R* rs7529229 with fatal and non-fatal coronary heart disease. Estimates are stratified by participant age, sex, the presence of CVD or diabetes at baseline, BMI, estimated risk of CVD based on the Framingham equation and by tertile of non-HDL-C and log CRP. The p-value from a chi-squared test of heterogeneity is also presented.

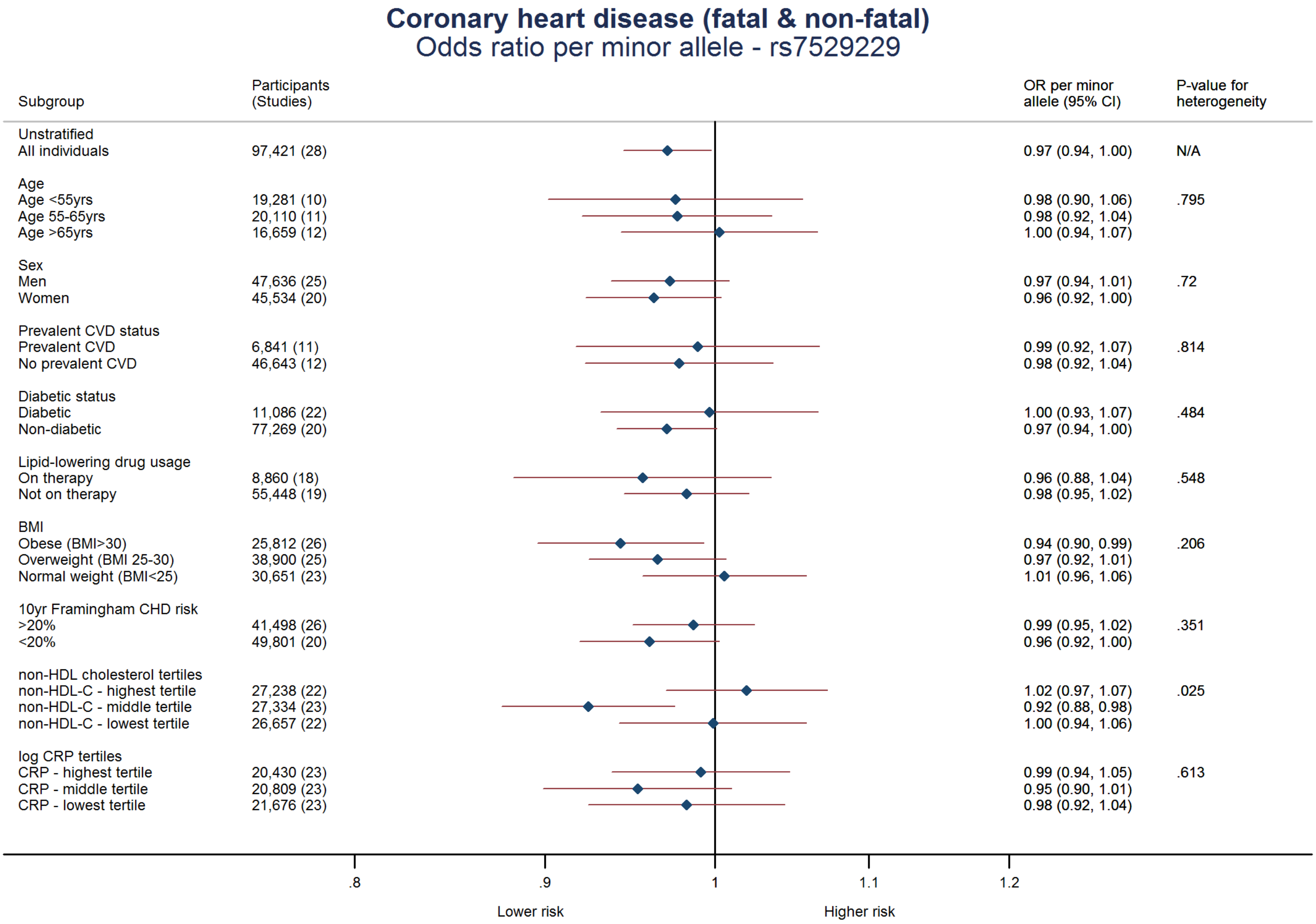

Supplementary Figure 10 - Association of *IL6R* rs7529229 with risk of CHD (fatal & non-fatal) stratified by genotyping platform used in each study contributing to the analysis

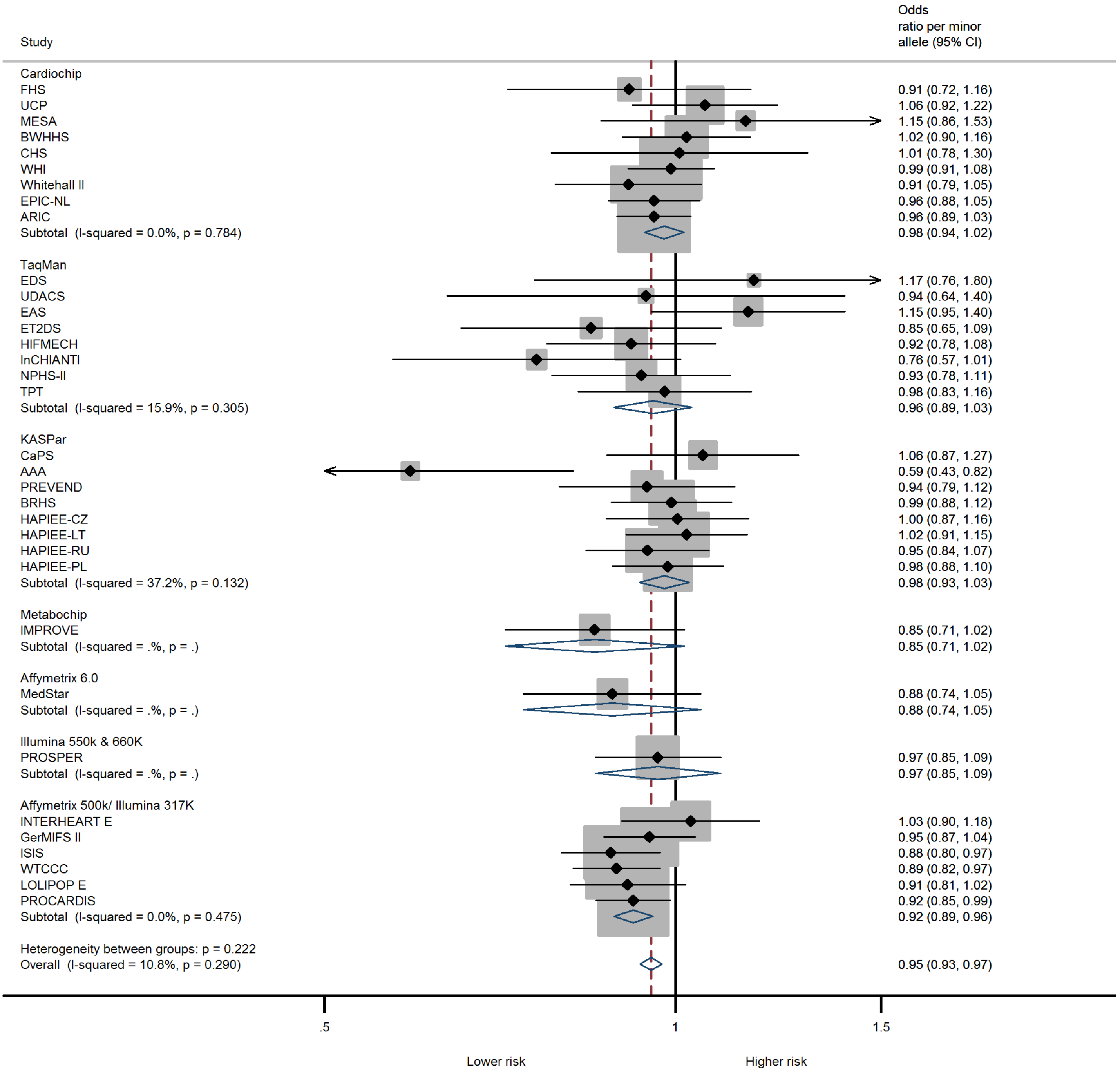

Supplementary references

1. Walker, M., Whincup, P.H. & Shaper, A.G. The British Regional Heart Study 1975-2004. *Int J Epidemiol* **33**, 1185-1192 (2004).
2. Lawlor, D.A., Bedford, C., Taylor, M. & Ebrahim, S. Geographical variation in cardiovascular disease, risk factors, and their control in older women: British Women's Heart and Health Study. *J Epidemiol Community Health* **57**, 134-140 (2003).
3. Caerphilly and Speedwell Collaborative Group Caerphilly and Speedwell collaborative heart disease studies. The Caerphilly and Speedwell Collaborative Group. *J Epidemiol Community Health* **38**, 259-262 (1984).
4. Fowkes, F.G. *et al.* Edinburgh Artery Study: prevalence of asymptomatic and symptomatic peripheral arterial disease in the general population. *Int J Epidemiol* **20**, 384-392 (1991).
5. Gardener, E.A., Huppert, F.A., Guralnik, J.M. & Melzer, D. Middle-aged and mobility-limited: prevalence of disability and symptom attributions in a national survey. *J Gen Intern Med* **21**, 1091-1096 (2006).
6. Price, J.F. *et al.* The Edinburgh Type 2 Diabetes Study: study protocol. *BMC Endocr Disord* **8**, 18 (2008).
7. Miller, G.J. *et al.* The effects of quality and timing of venepuncture on markers of blood coagulation in healthy middle-aged men. *Thromb. Haemost* **73**, 82-86 (1995).
8. Marmot, M. & Brunner, E. Cohort Profile: the Whitehall II study. *Int J Epidemiol* **34**, 251-256 (2005).
9. Wadsworth, M., Kuh, D., Richards, M. & Hardy, R. Cohort Profile: The 1946 National Birth Cohort (MRC National Survey of Health and Development). *Int J Epidemiol* **35**, 49-54 (2006).
10. Musunuru, K. *et al.* Candidate gene association resource (CARe): design, methods, and proof of concept. *Circ Cardiovasc Genet* **3**, 267-275 (2010).
11. ARIC Investigators The Atherosclerosis Risk in Communities (ARIC) Study: design and objectives. The ARIC investigators. *Am. J. Epidemiol* **129**, 687-702 (1989).
12. Friedman, G.D. *et al.* CARDIA: study design, recruitment, and some characteristics of the examined subjects. *J Clin Epidemiol* **41**, 1105-1116 (1988).
13. Redline, S. *et al.* The familial aggregation of obstructive sleep apnea. *Am. J. Respir. Crit. Care Med* **151**, 682-687 (1995).
14. Buxbaum, S.G., Elston, R.C., Tishler, P.V. & Redline, S. Genetics of the apnea hypopnea index in Caucasians and African Americans: I. Segregation analysis. *Genet. Epidemiol* **22**, 243-253 (2002).
15. Fried, L.P. *et al.* The Cardiovascular Health Study: design and rationale. *Ann Epidemiol* **1**, 263-276 (1991).
16. DAWBER, T.R., MEADORS, G.F. & MOORE, F.E., Jr Epidemiological approaches to heart disease: the Framingham Study. *Am J Public Health Nations Health* **41**, 279-281 (1951).
17. Bild, D.E. *et al.* Multi-ethnic study of atherosclerosis: objectives and design. *Am. J. Epidemiol* **156**, 871-881 (2002).
18. Peasey, A. *et al.* Determinants of cardiovascular disease and other non-communicable diseases in Central and Eastern Europe: rationale and design of the HAPIEE study. *BMC Public Health* **6**, 255 (2006).
19. Williams, D.R. *et al.* Undiagnosed glucose intolerance in the community: the Isle of Ely Diabetes Project. *Diabet. Med* **12**, 30-35 (1995).
20. Medical Research Council MRC Fenland Study. *MRC Fenland Study* (2010).at <http://www.mrc-epid.cam.ac.uk/Research/Studies/Fenland/index.html>
21. Baldassarre, D. *et al.* Cross-sectional analysis of baseline data to identify the major determinants of carotid intima-media thickness in a European population: the IMPROVE study. *Eur. Heart J* **31**, 614-622 (2010).
22. Diercks, G.F. *et al.* Rationale, design, and baseline characteristics of a trial of prevention of cardiovascular and renal disease with fosinopril and pravastatin in nonhypertensive, nonhypercholesterolemic subjects with microalbuminuria (the Prevention of REnal and Vascular ENdstage Disease Intervention Trial [PREVEND IT]). *Am. J. Cardiol* **86**, 635-638 (2000).
23. Simons, P.C., Algra, A., van de Laak, M.F., Grobbee, D.E. & van der Graaf, Y. Second manifestations of ARTerial disease (SMART) study: rationale and design. *Eur. J. Epidemiol* **15**, 773-781 (1999).
24. Ferrucci, L. *et al.* Subsystems contributing to the decline in ability to walk: bridging the gap between epidemiology and geriatric practice in the InCHIANTI study. *J Am Geriatr Soc* **48**, 1618-1625 (2000).
25. Reilly, M.P. *et al.* Identification of ADAMTS7 as a novel locus for coronary atherosclerosis and association of ABO with myocardial infarction in the presence of coronary atherosclerosis: two genome-wide association studies. *The Lancet* **377**, 383-392 (2011).
26. Fowkes, F.G.R. *et al.* Aspirin for prevention of cardiovascular events in a general population screened for a low ankle brachial index: a randomized controlled trial. *JAMA* **303**, 841-848 (2010).
27. Shepherd, J. *et al.* The design of a prospective study of Pravastatin in the Elderly at Risk (PROSPER). PROSPER Study Group. PROspective Study of Pravastatin in the Elderly at Risk. *Am. J. Cardiol* **84**, 1192-1197 (1999).
28. MRC GPRF Thrombosis prevention trial: randomised trial of low-intensity oral anticoagulation with warfarin and low-dose aspirin in the primary prevention of ischaemic heart disease in men at increased risk. The Medical Research Council's General Practice Research Framework. *Lancet* **351**, 233-241 (1998).
29. Beulens, J.W.J. *et al.* Cohort profile: the EPIC-NL study. *Int J Epidemiol* **39**, 1170-1178 (2010).
30. Day, N. *et al.* EPIC-Norfolk: study design and characteristics of the cohort. European Prospective Investigation of Cancer. *Br. J. Cancer* **80 Suppl 1**, 95-103 (1999).
31. Peters, B.J.M. *et al.* Genetic variability within the cholesterol lowering pathway and the effectiveness of statins in reducing the risk of MI. *Atherosclerosis* **217**, 458-464 (2011).

32. Anderson, G.L. *et al.* Implementation of the Women's Health Initiative study design. *Ann Epidemiol* **13**, S5-17 (2003).
33. Tang, T.S. *et al.* Association between the rs1050450 glutathione peroxidase-1 (C > T) gene variant and peripheral neuropathy in two independent samples of subjects with diabetes mellitus. *Nutr Metab Cardiovasc Dis* (2010).doi:10.1016/j.numecd.2010.08.001
34. Dhamrait, S.S. *et al.* Cardiovascular risk in healthy men and markers of oxidative stress in diabetic men are associated with common variation in the gene for uncoupling protein 2. *Eur. Heart J* **25**, 468-475 (2004).
35. Mannila, M.N. *et al.* Plasma fibrinogen concentration predicts the risk of myocardial infarction differently in various parts of Europe: effects of beta-fibrinogen genotype and environmental factors. The HIFMECH Study. *Thromb. Haemost* **92**, 1240-1249 (2004).
36. Meschia, J.F. *et al.* The Ischemic Stroke Genetics Study (ISGS) Protocol. *BMC Neurol* **3**, 4 (2003).
37. Meschia, J.F. *et al.* The Siblings With Ischemic Stroke Study (SWISS) protocol. *BMC Med. Genet* **3**, 1 (2002).
38. Tomlinson, I. *et al.* A genome-wide association scan of tag SNPs identifies a susceptibility variant for colorectal cancer at 8q24.21. *Nat. Genet* **39**, 984-988 (2007).
39. Schunkert, H. *et al.* Repeated replication and a prospective meta-analysis of the association between chromosome 9p21.3 and coronary artery disease. *Circulation* **117**, 1675-1684 (2008).
40. Pare, G. *et al.* Genetic analysis of 103 candidate genes for coronary artery disease and associated phenotypes in a founder population reveals a new association between endothelin-1 and high-density lipoprotein cholesterol. *Am. J. Hum. Genet* **80**, 673-682 (2007).
41. ISIS Collaborative Group ISIS-3: a randomised comparison of streptokinase vs tissue plasminogen activator vs anistreplase and of aspirin plus heparin vs aspirin alone among 41,299 cases of suspected acute myocardial infarction. ISIS-3 (Third International Study of Infarct Survival) Collaborative Group. *Lancet* **339**, 753-770 (1992).
42. Clarke, R. *et al.* Lymphotoxin-alpha gene and risk of myocardial infarction in 6,928 cases and 2,712 controls in the ISIS case-control study. *PLoS Genet* **2**, e107 (2006).
43. Chambers, J.C. *et al.* Common genetic variation near MC4R is associated with waist circumference and insulin resistance. *Nat. Genet* **40**, 716-718 (2008).
44. Farrall, M. *et al.* Genome-wide mapping of susceptibility to coronary artery disease identifies a novel replicated locus on chromosome 17. *PLoS Genet* **2**, e72 (2006).
45. Samani, N.J. *et al.* Genomewide association analysis of coronary artery disease. *N. Engl. J. Med* **357**, 443-453 (2007).
46. Johnson, N. *et al.* Interaction between CHEK2*1100delC and other low-penetrance breast-cancer susceptibility genes: a familial study. *Lancet* **366**, 1554-1557 (2005).
47. Stacey, S.N. *et al.* Common variants on chromosomes 2q35 and 16q12 confer susceptibility to estrogen receptor-positive breast cancer. *Nat. Genet* **39**, 865-869 (2007).
48. Power, C. & Elliott, J. Cohort profile: 1958 British birth cohort (National Child Development Study). *Int J Epidemiol* **35**, 34-41 (2006).
49. Fletcher, O. *et al.* Novel breast cancer susceptibility locus at 9q31.2: results of a genome-wide association study. *J. Natl. Cancer Inst* **103**, 425-435 (2011).
50. Friedewald, W.T., Levy, R.I. & Fredrickson, D.S. Estimation of the concentration of low-density lipoprotein cholesterol in plasma, without use of the preparative ultracentrifuge. *Clin. Chem* **18**, 499-502 (1972).
51. International HapMap Consortium The International HapMap Project. *Nature* **426**, 789-796 (2003).
52. Barrett, J.C., Fry, B., Maller, J. & Daly, M.J. Haploview: analysis and visualization of LD and haplotype maps. *Bioinformatics* **21**, 263-265 (2005).
53. Kent, W.J. *et al.* The human genome browser at UCSC. *Genome Res* **12**, 996-1006 (2002).
54. Keating, B.J. *et al.* Concept, design and implementation of a cardiovascular gene-centric 50 k SNP array for large-scale genomic association studies. *PLoS ONE* **3**, e3583 (2008).
55. Singh, J.A., Beg, S. & Lopez-Olivo, M.A. Tocilizumab for rheumatoid arthritis. *Cochrane Database Syst Rev* CD008331 (2010).doi:10.1002/14651858.CD008331.pub2
56. Elliott, P. *et al.* Genetic Loci associated with C-reactive protein levels and risk of coronary heart disease. *JAMA* **302**, 37-48 (2009).
57. Melzer, D. *et al.* A genome-wide association study identifies protein quantitative trait loci (pQTLs). *PLoS Genet* **4**, e1000072 (2008).
58. Kibriya, M.G. *et al.* A pilot genome-wide association study of early-onset breast cancer. *Breast Cancer Res. Treat* **114**, 463-477 (2009).
59. Heikkilä, K. *et al.* C-reactive protein-associated genetic variants and cancer risk: findings from FINRISK 1992, FINRISK 1997 and Health 2000 studies. *Eur. J. Cancer* **47**, 404-412 (2011).
60. Li, J. *et al.* A combined analysis of genome-wide association studies in breast cancer. *Breast Cancer Res. Treat* **126**, 717-727 (2011).
61. Rantala, A. *et al.* Association of IL-6 and IL-6R gene polymorphisms with susceptibility to respiratory tract infections in young Finnish men. *Hum. Immunol* **72**, 63-68 (2011).
62. Smolen, J.S. *et al.* Effect of interleukin-6 receptor inhibition with tocilizumab in patients with rheumatoid arthritis (OPTION study): a double-blind, placebo-controlled, randomised trial. *Lancet* **371**, 987-997 (2008).

63. Genovese, M.C. *et al.* Interleukin-6 receptor inhibition with tocilizumab reduces disease activity in rheumatoid arthritis with inadequate response to disease-modifying antirheumatic drugs: the tocilizumab in combination with traditional disease-modifying antirheumatic drug therapy study. *Arthritis Rheum* **58**, 2968-2980 (2008).
64. Emery, P. *et al.* IL-6 receptor inhibition with tocilizumab improves treatment outcomes in patients with rheumatoid arthritis refractory to anti-tumour necrosis factor biologicals: results from a 24-week multicentre randomised placebo-controlled trial. *Ann. Rheum. Dis* **67**, 1516-1523 (2008).
65. Kremer, J.L. *et al.* Tocilizumab inhibits structural joint damage in rheumatoid arthritis patients with inadequate responses to methotrexate at 1 year: The LITHE study. *Arthritis Rheum* (2010).doi:10.1002/art.30158

Name	Affil 1	Affil2	Affil 3	Qualifications
Dr Daniel I Swerdlow	Research Department of Epidemiology and Public Health, University College London, London, UK			PhD
Dr Michael V Holmes	Research Department of Epidemiology and Public Health, University College London, London, UK			MSc DLSHTM MBBS MRCP
Ms Karoline B Kuchenbaecker	Centre for Cancer Genetic Epidemiology, Department of Public Health and Primary Care, University of Cambridge, Cambridge, UK			Dipl.-Psych., M.Sc. Biom.
Mr Jorgen EL Engmann	Research Department of Epidemiology and Public Health, University College London, London, UK			MSc BSc
Dr Tina Shah	Research Department of Epidemiology and Public Health, University College London, London, UK	Centre for Clinical Pharmacology, Division of Medicine, University College London, London, UK		PhD
Dr Reecha Sofat	Research Department of Epidemiology and Public Health, University College London, London, UK	Centre for Clinical Pharmacology, Division of Medicine, University College London, London, UK		PhD
Dr Yiran Guo	Center for Applied Genomics, Abramson Research Center, The Children's Hospital of Philadelphia, Philadelphia, Pennsylvania 19104, USA	BGI-Shenzhen, Beishan Industrial Zone, Yantian District, Shenzhen 518083, China		PhD
Ms Christina Chung	Research Department of Epidemiology and Public Health, University College London, London, UK			MSc
Dr Anne Peasey	Research Department of Epidemiology and Public Health, University College London, London, UK			PhD
Dr Roman Pfister	MRC Epidemiology Unit, Institute of Metabolic Science, University of Cambridge	Department III of Internal Medicine, University of Cologne, Cologne, Germany		MD
Dr Simon P Mooijaart	Department of Gerontology and Geriatrics , Leiden University Medical Center			MD, PhD
Dr Helen A Ireland	Institute of Cardiovascular Science, UCL, London, UK			PhD
Mr Maarten Leusink	Division of Pharmacoepidemiology and Clinical Pharmacology, Utrecht Institute for Pharmaceutical Sciences, Faculty of Science, Utrecht University, Utrecht, the Netherlands			MSc
Dr Claudia Langenberg	MRC Epidemiology Unit, Institute of Metabolic Science, University of Cambridge			MD PhD
Ms KaWah Li	Centre for Cardiovascular Genetics, Institute of Cardiovascular Science, University College London, 5 University Street, London WC1E 6JF, UK			MSc
Ms Jutta Palmen	Centre for Cardiovascular Genetics, Institute of Cardiovascular Science, University College London, 5 University Street, London WC1E 6JF, UK			
Mr Philip Howard	Centre for Cardiovascular Genetics, Institute of Cardiovascular Science, University College London, 5 University Street, London WC1E 6JF, UK			
Ms Jackie A Cooper	Centre for Cardiovascular Genetics, Institute of Cardiovascular Science, University College London, 5 University Street, London WC1E 6JF, UK			MSc
Dr Fotios Drenos	Centre for Cardiovascular Genetics, Institute of Cardiovascular Science, University College London, 5 University Street, London WC1E 6JF, UK			PhD
Professor John Hardy	Reta Lila Weston Institute of Neurological Studies, Institute of Neurology, UCL, London, UK			PhD, MD (Hon), DSc, FMedSci, FRS
Dr Michael A Nalls	National Institute on Aging, National Institutes of Health, Bethesda, MD 20892			PhD
Ms Yun Rose Li	Center for Applied Genomics, Abramson Research Center, The Children's Hospital of Philadelphia, Philadelphia, Pennsylvania 19104, USA			BSc
Professor Gordon Lowe	Institute of Cardiovascular and Medical Sciences, University of Glasgow			DSc
Dr Marlene Stewart	University of Edinburgh			PhD
Dr Suzette J Bielinski	Division of Epidemiology, Department of Health Sciences Research, Mayo Clinic, Harwick Building 6-56, 200 First Street South West, Rochester, MN, 55909, USA			
Professor Julian Peto	Department of non-communicable disease epidemiology, London School of Hygiene and Tropical Medicine, London WC1E 7HT			DSc
Dr Nicholas J Timpson	MRC Centre for Causal Analyses in Translational Epidemiology, University of Bristol	School of Social and Community Medicine, University of Bristol		PhD

Dr John Gallacher	Institute of Primary Care and Public Health, Cardiff University, Cardiff, UK		
Professor Malcolm Dunlop	MRC Human Genetics Unit, Western General Hospital, Edinburgh		MD, FRCS, FMedSci, FRSE
Professor Richard Houlston	Section of Cancer Genetics, Institute of Cancer Research		
Professor Ian Tomlinson	Wellcome Trust Centre for Human Genetics and NIHR Comprehensive Biomedical Research Centre		
Dr Ioanna Tzoulaki	Department of Epidemiology and Biostatistics, Imperial College London, London, UK	Department of Hygiene and Epidemiology, University of Ioannina Medical School, Ioannina, Greece	PhD
Dr Jian'an Luan	MRC Epidemiology Unit, Institute of Metabolic Science, University of Cambridge		PhD
Dr Jolanda MA Boer	National Institute for Public Health and the Environment, Bilthoven, the Netherlands		PhD
Dr Nita G Forouhi	MRC Epidemiology Unit, Institute of Metabolic Science, Box 285, Addenbrooke's Hospital, Hills Road, Cambridge CB2 0QQ, UK		MRCP, PhD, FFPH
Dr N Charlotte Onland-Moret	Julius Center for Health Sciences and Primary Care, University Medical Center, Utrecht, Utrecht, The Netherlands		
Professor Yvonne T van der Schouw	Julius Center for Health Sciences and Primary Care, University Medical Center, Utrecht, Utrecht, The Netherlands		
Dr Renate Schnabel	University Heart Center Hamburg, Department of General and Interventional Cardiology, Hamburg, Germany		MD, MSc
Dr Jaroslav A Hubacek	Institute for Clinical and Experimental Medicine, Prague, Czech Republic		PhD, DSc
Dr Ruzena Kubinova	Department of Environmental Health Monitoring System, National Institute of Public Health, Prague, Czech Republic		MD
Dr Migle Baceviciene	Lithuanian University of Health Sciences, Kaunas, Lithuania		MD, PhD
Professor Abdonas Tamosiunas	Lithuanian University of Health Sciences, Kaunas, Lithuania		MD
Prof Andrzej Pajak	Institute of Public Health, Jagiellonian University Medical College, Krakow, Poland		MD, PhD
Dr Roman Topor-Madry	Institute of Public Health, Jagiellonian University Medical College, Krakow, Poland		MD, PhD
Professor Sofia Malyutina	Institute of Internal Medicine SB RAMS, Novosibirsk, Russia		MD, PhD
Dr Damiano Baldassarre	Department of Pharmacological Sciences, University of Milan, Monzino Cardiology Center, IRCCS, Milan Italy		PhD
Dr Bengt Sennblad	Atherosclerosis Research Unit, Department of Medicine Solna, Karolinska Institutet, Stockholm, Sweden		PhD
Professor Elena Tremoli	Department of Pharmacological Sciences, University of Milan, Monzino Cardiology Center, IRCCS, Milan Italy		PhD
Professor Ulf de Faire	Division of Cardiovascular Epidemiology, Institute of Environmental Medicine, Karolinska Institutet, Stockholm, Sweden		MD PhD
Dr Luigi Ferrucci	National Institute on Aging, Clinical Research Branch , Harbor Hospital, Hanover Street, Baltimore, MD, USA		MD, PhD
Dr Stefania Bandenelli	Florence Local Health Agency (ASF-Firenze), Florence, Italy		MD
Dr Toshiko Tanaka	National Institute on Aging, Clinical Research Branch , Harbor Hospital, Hanover Street, Baltimore, MD, USA		PhD
Dr James F Meschia	Department of Neurology, Mayo Clinic, Jacksonville, FL, USA		MD
Dr Andrew Singleton	National Institute on Aging, National Institutes of Health, Bethesda, MD 20892		PhD
Professor Gerjan Navis	Department of Internal Medicine University Medical Center Groningen, University of Groningen, Groningen, The Netherlands		MD, PhD
Dr Irene Mateo Leach	Department of Cardiology, University Medical Center Groningen, University of Groningen, Groningen, The Netherlands		PhD

Dr Stephan JL Bakker	Department of Internal Medicine University Medical Center Groningen, University of Groningen, Groningen, The Netherlands		MD, PhD
Dr Ron T Gansevoort	Department of Nephrology, University Medical Center Groningen, University of Groningen, Groningen, The Netherlands		MD, PhD
Professor Ian Ford	Centre for Biostatistics, University of Glasgow		DSc
Dr. Stephen E Epstein	Cardiovascular Research Institute, MedStar Health Research Institute, Washington Hospital Center, Washington, DC, USA		MD
Dr. Mary Susan Burnett	Cardiovascular Research Institute, MedStar Health Research Institute, Washington Hospital Center, Washington, DC, USA		PhD
Dr. Joe M Devaney	Cardiovascular Research Institute, MedStar Health Research Institute, Washington Hospital Center, Washington, DC, USA		PhD
Professor J Wouter Jukema	Dept of Cardiology C5-P Leiden University Medical Center		FACC
Professor Rudi GJ Westendorp	Department of Gerontology and Geriatrics , Leiden University Medical Center, The Netherlands		MD, PhD
Dr Gert Jan de Borst	Department of Vascular Surgery, University Medical Center Utrecht, Utrecht, The Netherlands		MD, PhD
Professor Yolanda van der Graaf	Julius Center for Health Sciences and Primary Care, University Medical Center Utrecht, Utrecht, The Netherlands		MD, PhD
Dr Pim A de Jong	Department of Radiology, University Medical Center Utrecht, Utrecht, The Netherlands		MD, PhD
Dr Anke-Hilse Maitland-van der Zee	Division of Pharmacoepidemiology and Clinical Pharmacology, Utrecht Institute for Pharmaceutical Sciences, Faculty of Science, Utrecht University, Utrecht, the Netherlands		PharmD, PhD
Dr Olaf H Klungel	Division of Pharmacoepidemiology and Clinical Pharmacology, Utrecht Institute for Pharmaceutical Sciences, Faculty of Science, Utrecht University, Utrecht, the Netherlands		PharmD, PhD
Professor Anthonius de Boer	Division of Pharmacoepidemiology and Clinical Pharmacology, Utrecht Institute for Pharmaceutical Sciences, Faculty of Science, Utrecht University, Utrecht, the Netherlands		MD, PhD
Professor Pieter A Doevendans	Department of Cardiology, Division Heart and Lungs , University Medical Centre Utrecht, Utrecht, The Netherlands		MD, PhD
Dr Jeffrey W Stephens	College of Medicine, Swansea University, Swansea SA2 8PP		MB, PhD, FRCP
Professor Charles B Eaton	Memorial Hospital of Rhode Island	Brown University	MD, MS
Professor Jennifer G Robinson	University of Iowa		MD, MPH
Professor JoAnn E Manson	Division of Preventive Medicine, Brigham and Women's Hospital		MD, DrPH
Professor F Gerry R Fowkes	University of Edinburgh		PhD,FRCPE
Professor Timothy M Frayling	Peninsula College of Medicine and Dentistry, University of Exeter, Exeter, UK		PhD
Dr Jackie Price	Centre for Population Health Sciences, University of Edinburgh, Edinburgh, UK		MD, FFPH
Professor Peter H Whincup	St George's, University of London		PhD FRCP
Professor Richard W Morris	Department of Primary Care & Population Health, UCL, London, UK		PhD
Professor Debbie A Lawlor	MRC Centre for Causal Analyses in Translational Epidemiology, University of Bristol	School of Social and Community Medicine, University of Bristol	PhD
Professor George Davey Smith	MRC Centre for Causal Analyses in Translational Epidemiology, University of Bristol	School of Social and Community Medicine, University of Bristol	
Professor Yoav Ben-Shlomo	School of Social and Community Medicine, University of Bristol		MB BS, PhD
Professor Susan Redline	Harvard Medical School, Brigham and Women's Hospital, 221 Longwood Ave Boston MA 02115		MD, MPH
Dr Leslie A Lange	Department of Genetics, University of North Carolina School of Medicine at Chapel Hill, Chapel Hill, North Carolina 27514, USA		

Dr Meena Kumari	Research Department of Epidemiology and Public Health, University College London, London, UK			PhD
Professor Nick J Wareham	MRC Epidemiology Unit, Institute of Metabolic Science, University of Cambridge			
Dr W M Monique Verschuren	National Institute for Public Health and the Environment, Bilthoven, the Netherlands			
Professor Emelia J Benjamin	Boston University Schools of Medicine and Public Health, Boston, MA.			
Professor John C Whittaker	GlaxoSmithKline	Faculty of Epidemiology and Population Health, London School of Hygiene and Tropical Medicine		PhD
Professor Anders Hamsten	Atherosclerosis Research Unit, Department of Medicine Solna, Karolinska Institutet, Stockholm, Sweden			MD PhD FRCP
Dr Frank Dudbridge	Faculty of Epidemiology and Population Health, London School of Hygiene and Tropical Medicine			PhD
Dr JA Chris Delaney	Department of Pharmaceutical Outcomes& Policy, College of Pharmacy, University of Florida, Gainesville, FL 32610-0496, USA			
Dr Andrew Wong	MRC Unit for Lifelong Health & Ageing, London, UK			PhD
Professor Diana Kuh	MRC Unit for Lifelong Health & Ageing, London, UK			PhD, FFPH
Dr Rebecca Hardy	MRC Unit for Lifelong Health & Ageing, London, UK			PhD
Dr Berta Almoguera Castillo	Center for Applied Genomics, Abramson Research Center, The Children's Hospital of Philadelphia, Philadelphia, Pennsylvania 19104, USA			
Dr John J Connolly	Center for Applied Genomics, Abramson Research Center, The Children's Hospital of Philadelphia, Philadelphia, Pennsylvania 19104, USA			
Dr Pim van der Harst	Department of Cardiology, University Medical Center Groningen, University of Groningen, Groningen, The Netherlands	Department of Genetics, University Medical Center Groningen, University of Groningen, Groningen, The Netherlands		MD, PhD
Dr Eric J Brunner	Research Department of Epidemiology and Public Health, University College London, London, UK			
Professor Sir Michael G Marmot	UCL Institute of Health Equity, Research Department of Epidemiology and Public Health, University College London, London, UK			FRCP
Professor Christina L Wassel	University of California - San Diego, Department of Family and Preventive Medicine			PhD
Professor Steve E Humphries	Centre for Cardiovascular Genetics, Institute of Cardiovascular Science, University College London, 5 University Street, London WC1E 6JF, UK			PhD MRCpath MRCP FAMS
Professor Philippa J Talmud	Centre for Cardiovascular Genetics, Institute of Cardiovascular Science, University College London, 5 University Street, London WC1E 6JF, UK			PhD, DSc, MRCPath
Professor Mika Kivimaki	Research Department of Epidemiology and Public Health, University College London, London, UK			PhD
Dr Folkert W Asselbergs	Department of Cardiology, Division Heart and Lungs, University Medical Center Utrecht, Utrecht, The Netherlands	Julius Center for Health Sciences and Primary Care, University Medical Center Utrecht, The Netherlands	Department of Medical Genetics, Biomedical Genetics, University Medical Center, Utrecht, The Netherlands	MD PhD
Professor Mikhail Voevoda	Institute of Internal Medicine SB RAMS, Novosibirsk, Russia			MD, PhD
Professor Martin Bobak	Research Department of Epidemiology and Public Health, University College London, London, UK			MD, PhD
Dr Hynek Pikhart	Research Department of Epidemiology and Public Health, University College London, London, UK			PhD
Professor James G Wilson	Department of Physiology and Biophysics, University of Mississippi Medical Center, Jackson, MS 39216 USA			
Professor Hakon Hakonarson	Center for Applied Genomics, Abramson Research Center, The Children's Hospital of Philadelphia, Philadelphia, Pennsylvania 19104, USA			

Professor Alex P Reiner	Division of Public Health Sciences, Fred Hutchinson Cancer Research Center, Seattle, Washington		MD, MS
Dr Brendan J Keating	Center for Applied Genomics, Abramson Research Center, The Children's Hospital of Philadelphia, Philadelphia, Pennsylvania 19104, USA		PhD
Professor Naveed Sattar	Institute of Cardiovascular & Medical Sciences, University of Glasgow		FRCP
Professor Aroon D Hingorani*	Research Department of Epidemiology and Public Health, University College London, London, UK		FRCP
Dr Juan Pablo Casas*	Research Department of Epidemiology and Public Health, University College London, London, UK	Department of Epidemiology and Population Health, London School of Hygiene and Tropical Medicine, London, UK	PhD

***Joint senior authors**
